# Type I interferon limits mast cell–mediated anaphylaxis by controlling secretory granule homeostasis

**DOI:** 10.1371/journal.pbio.3000530

**Published:** 2019-11-15

**Authors:** Toshihiko Kobayashi, Shiho Shimabukuro-Demoto, Hidemitsu Tsutsui, Noriko Toyama-Sorimachi

**Affiliations:** Department of Molecular Immunology and Inflammation, Research Institute, National Center for Global Health and Medicine, Shinjuku-ku, Tokyo, Japan; TUM School of Medicine, GERMANY

## Abstract

Type I interferon (IFN-I) is a family of multifunctional cytokines that modulate the innate and adaptive immunity and are used to treat mastocytosis. Although IFN-I is known to suppress mast cell function, including histamine release, the mechanisms behind its effects on mast cells have been poorly understood. We here investigated IFN-I’s action on mast cells using interferon-α/β receptor subunit 1 (*Ifnar1*)-deficient mice, which lack a functional IFN-I receptor complex, and revealed that IFN-I in the steady state is critical for mast cell homeostasis, the disruption of which is centrally involved in systemic anaphylaxis. *Ifnar1*-deficient mice showed exacerbated systemic anaphylaxis after sensitization, which was associated with increased histamine in the circulation, even though the mast cell numbers and high affinity immunoglobulin E receptor (FcεRI) expression levels were similar between *Ifnar1*-deficient and wild-type (WT) mice. *Ifnar1*-deficient mast cells showed increased secretory granule synthesis and exocytosis, which probably involved the increased transcription of *Tfeb*. Signal transducer and activator of transcription 1(Stat1) and Stat2 were unexpectedly insufficient to mediate these IFN-I functions, and instead, Stat3 played a critical role in a redundant manner with Stat1. Our findings revealed a novel regulation mechanism of mast cell homeostasis, in which IFN-I controls lysosome-related organelle biogenesis.

## Abstract 2

This study reveals a novel role for type I interferon in mast cell homeostasis; spontaneous type I interferon signaling regulates the biogenesis of secretory granules and maturation of mast cells via STAT1 and STAT3, and limits the onset of systemic anaphylaxis.

## Introduction

Type I interferon (IFN-I) is a family of pleiotropic cytokines that modulate the innate and adaptive immunity and play a critical role in antiviral responses [1]. The IFN-I family consists of 13 IFN-α subtypes and IFN-β, IFN-β, IFN-κ, and IFN-ω, and others in some species [2,3]. All IFN-I molecules bind to a receptor consisting of type-I interferon (IFN-α/β) receptor (IFNAR)1 and IFNAR2, and the resulting ternary complex forms an active signaling receptor [4]. Although IFN-I interacts with IFNAR1 and IFNAR2 with different affinities, structural analyses combined with site-directed mutagenesis demonstrated that IFNAR1 is necessary for the signaling of IFN-I [2,5]. After IFN-I binds to the receptor, the Janus family kinases (Jak) tyrosine kinase 2 (Tyk2) and Jak1 are activated by reciprocal transphosphorylation [6]. These activated kinases then mediate the transcription of a large number of genes, the so-called IFN-stimulated genes (ISGs), through the phosphorylation-dependent activation of the transcription factors, signal transduction and activator of transcription (STAT)1 and STAT2 [6,7]. This Jak-Stat pathway downstream of IFNAR is critical for the activation of various types of immune cells, which shapes the adaptive immunity, and therefore disorders observed in IFNAR1 or IFNAR2 deficiency largely overlap those observed in STAT1 or STAT2 deficiency [8,9]. IFN-I is also known to activate STAT3 through IFNAR, which confers negative effects on ISG expression [10–13], but the physiological role of STAT3 in IFN signaling is still largely unclear.

IFN-I has been clinically used to treat mastocytosis [14–16]. This rare and intractable disease is primarily caused by disordered mast cell proliferation and activation, and exhibits diverse symptoms, including pruritus, flushing, and anaphylactic shock [15]. Many of these symptoms are due to mediators released from mast cells, such as histamine [14]. IFN-I treatment ameliorates the symptoms by inhibiting mast cell proliferation, degranulation, bone marrow infiltration, or mastocytosis-related ascites/hepatosplenomegaly [17–19]. Within this context, studies using neoplastic mast cell lines and cultured bone marrow–derived mast cells (BMMCs) have been performed and showed that IFN-I suppresses the histamine release from mast cells [15,20–22]. However, the molecular mechanisms by which IFN-I affects mast cell functions are not well understood.

Mast cells are key players in the pathogenesis of allergic responses. These cells differentiate in the bone marrow and reside in peripheral tissues, where they functionally adapt to their environment and undergo terminal maturation. Mast cells secrete their granule contents and/or cytokines when they are activated by stimuli such as high affinity immunoglobulin E receptor (FcεRI) cross-linking by an antigen bound to its specific immunoglobulin E (IgE). The mast cell’s effector functions depend largely on the well-developed lysosome-related organelles (LROs), known as secretory granules [23], which contain histamine, proteases, and other inflammatory mediators [24]. Although the precise mechanisms of LRO biogenesis and functional adaptation are still largely unclear, one of the key molecules involved is the transcription factor EB (TFEB) [25]. TFEB regulates a set of gene expressions involved in lysosome biogenesis, known as the coordinated lysosomal expression and regulation (CLEAR) gene network, and is considered the master regulator of lysosomal biogenesis [26,27]. TFEB’s transcriptional activity is regulated by the mechanistic target of rapamycin complex 1 (mTORC1) pathway, in which TFEB’s nuclear translocation is controlled by its cytosolic sequestration, which is regulated by mTORC1-mediated phosphorylation [28,29]. In addition, TFEB’s expression levels are positively correlated with its transcriptional activity, because TFEB induces its own transcription [26]. Furthermore, the increase in TFEB protein boosts its nuclear translocation. We previously reported that mast cell secretory granules are also under the control of TFEB in a manner depending on the lysosomal environment, which is regulated by a lysosome-resident amino acid transporter [30].

Here, we used IFNAR1-deficient mice to investigate how IFN-I influences mast cells, and revealed a critical role of IFN-I in controlling mast cell homeostasis in the steady state. We found that the loss of steady-state IFN-I signaling caused dysregulated secretory granule formation, which strongly promoted the onset of systemic anaphylaxis. Unexpectedly, STAT1 and STAT2 were not sufficient to elicit this IFN-I function, and instead, STAT3 played a critical role, in a manner redundant with STAT1. Our findings reveal new information about the action of IFN-I in mast cells and provide important insight for therapeutic strategies for mast cell–mediated allergic reactions.

## Results

### IFN-I signaling limits IgE-mediated systemic and local anaphylaxis

**Fig 1:**
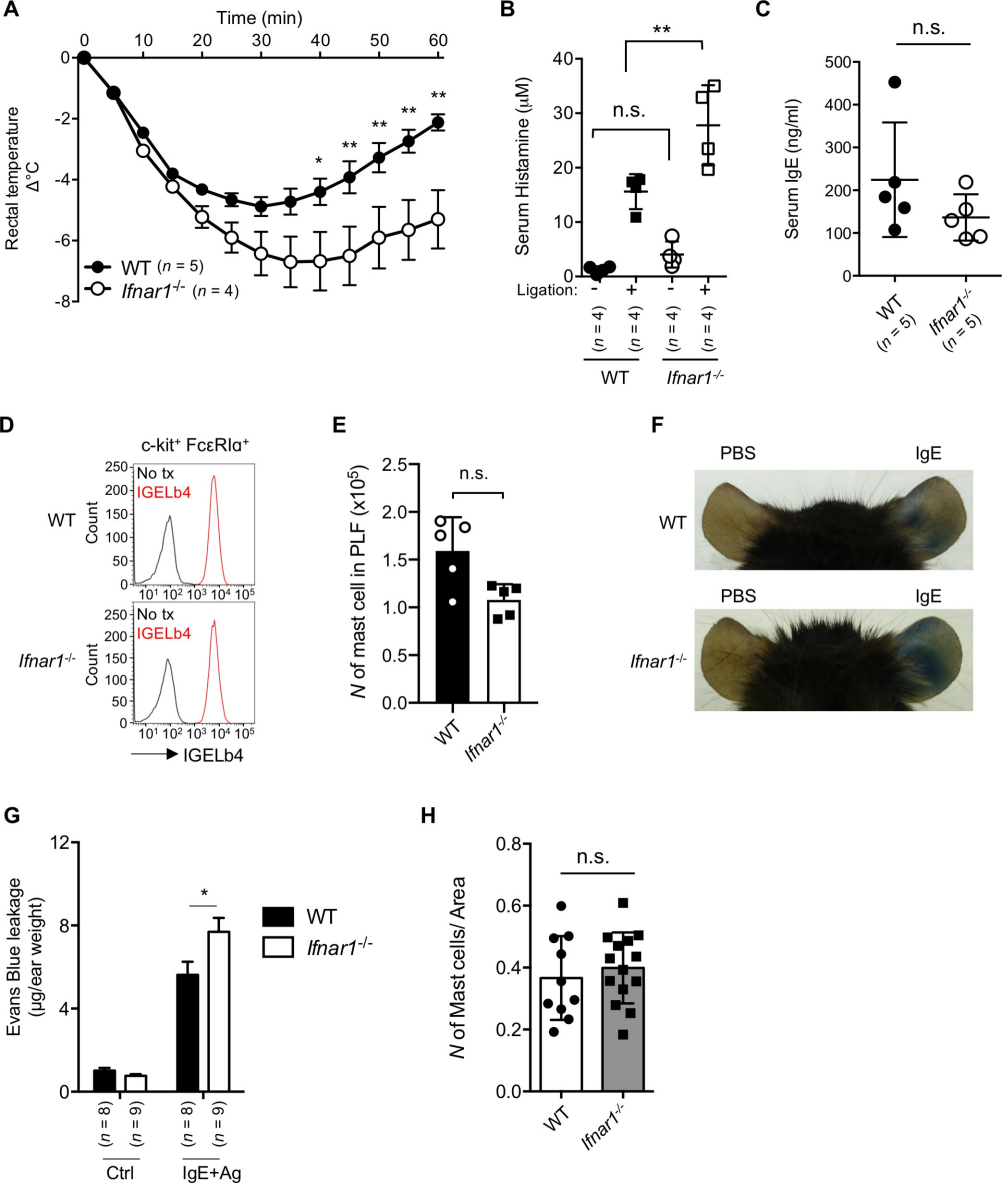
IFNAR-mediated signaling reduced IgE-mediated anaphylaxis. (A) IgE-mediated passive systemic anaphylaxis. WT or *Ifnar1*^−/−^ mice sensitized with anti-TNP IgE were challenged with TNP_4_-BSA, and the body temperature was monitored for 60 minutes. *n* = 5 for WT; *n* = 4 for *Ifnar1*^−/−^. **P < 0.01. Results are shown as the mean ± SEM of values representative of three independent experiments. (B) Quantification of serum histamine upon FcεRI ligation. *n* = 4 for each group. n.s., not significant, **P < 0.01. Results are shown as the mean ± SD of values representative of three independent experiments. (C) Serum IgE level in naïve WT and *Ifnar1*^−/−^ mice. Dots indicate individual mice. *n* = 5 for each group. n.s., not significant. Results are shown as the mean ± SD of values representative of three independent experiments. (D) Exogenous IgE binding in vivo on peritoneal mast cells of individual mouse analyzed by flow cytometry. Data are representative of three independent experiments. (E) Number of peritoneal mast cells in naïve mice in the peritoneal lavage fluid. *n* = 5 for each group. n.s., not significant. (F) Representative images of Evans blue leakage upon PCA. (G) Quantification of Evans blue leakage during PCA. *n* = 8 for WT; *n* = 9 for *Ifnar1*^−/−^ mice. *P < 0.05. (H) Number of cutaneous mast cells in naïve mice. The number of mast cells in a defined area of the ear (200 μm^2^) was counted; *n* = 10 for WT and *n* = 14 for *Ifnar1*^−/−^ mice. n.s., not significant. Underlying data in A, B, C, E, G, and H can be found in S1 Data. BSA, bovine serum albumin; FcεRI, high affinity immunoglobulin E receptor; IFNAR, Interferon-α/β receptor; IgE, immunoglobulin E; IGELb4, Anti-TNP IgE monoclonal antibody (clone IGELb4); PCA, passive cutaneous anaphylaxis; TNP, trinitrophenyl; WT, wild-type.

To investigate the mechanism by which IFN-I affects mast cell functions, we first examined the mast cell–dependent systemic anaphylaxis using IFNAR1-deficient mice (*Ifnar1*^*−/−*^). When mice were passively inoculated with trinitrophenyl (TNP)–specific IgE and TNP-conjugated bovine serum albumin (BSA), more severe hypothermia was observed in the *Ifnar1*^*−/−*^ mice than in the wild-type (WT) mice (Fig 1A). Consistent with this observation, the serum histamine levels after FcεRI cross-linking were significantly higher in the *Ifnar1*^*−/−*^ mice than in WT mice (Fig 1B). These results were unexpected, because even though the mice did not receive exogenously administered IFN-I or agonistic reagents to induce IFN-I production, the IFNAR1 loss strongly influenced the onset of systemic anaphylaxis. The serum IgE concentration at steady state was similar between the *Ifnar1*^*−/−*^ and WT mice (Fig 1C), as was the binding of passively administered antigen-specific IgE to mast cells (Fig 1D). The peritoneal mast cell numbers were not significantly different between WT and *Ifnar1*^*−/−*^ mice (Fig 1E). We also examined local anaphylaxis using the passive cutaneous anaphylaxis (PCA) model and found that the endothelial permeability was increased in the *Ifnar1*^*−/−*^ mice (Fig 1F and 1G). The number of ear-resident mast cells was similar between WT and *Ifnar1*^*−/−*^ mice (Fig 1H). These results indicated that IFNAR1 plays a critical role in limiting mast cell–dependent anaphylaxis.

### Mast cells respond to IFN-I in autocrine and paracrine manners

**Fig 2:**
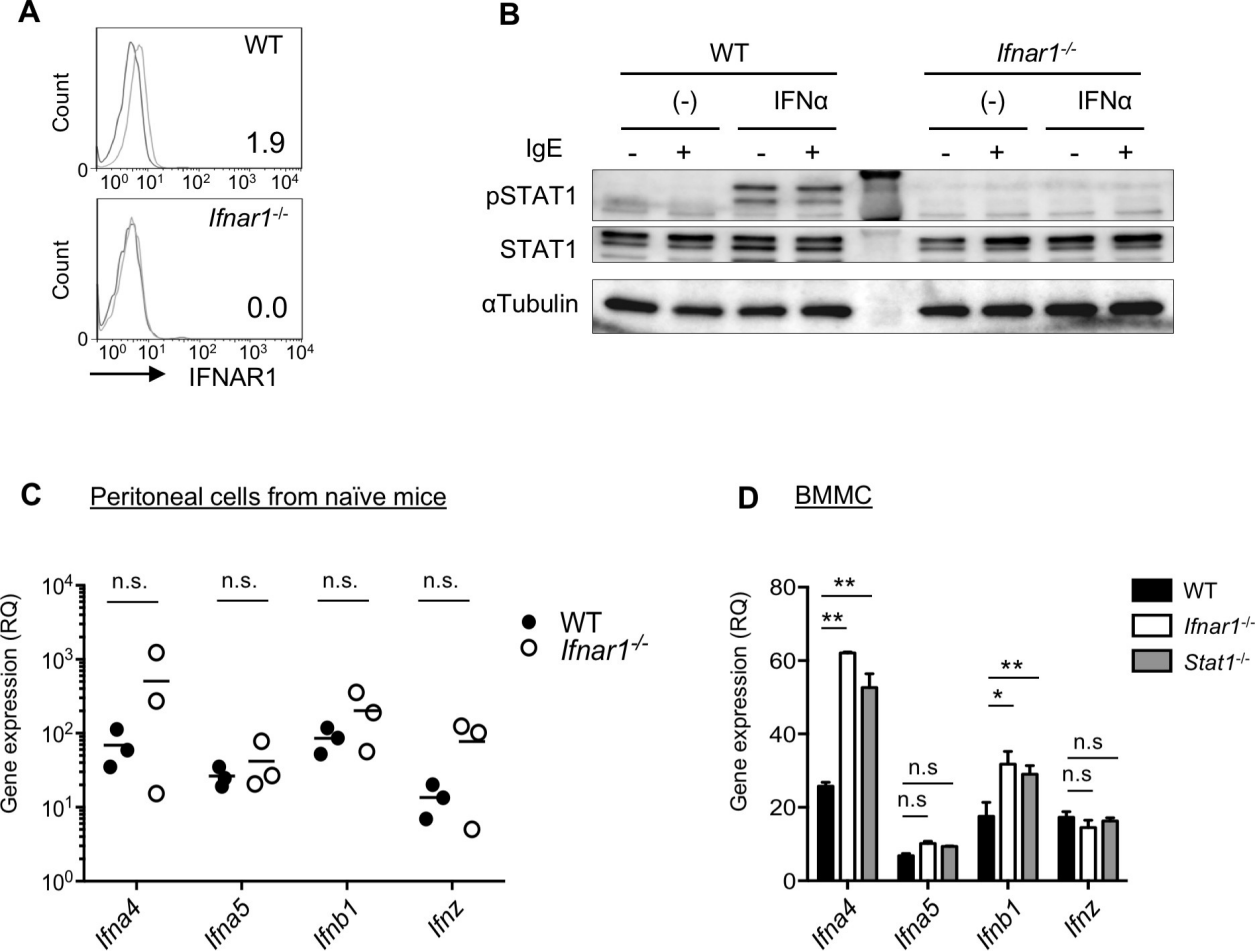
Mast cells express IFNAR and can respond to IFN-I probably produced in their niche. (A) IFNAR1 expressed on the surface of the peritoneal mast cells was detected by flow cytometry. The mean fluorescent intensity after subtracting the background is indicated. (B) IFN-I signaling in BM-derived mast cells. WT or *Ifnar1*^−/−^ BM-derived mast cells were treated with recombinant IFN-α for 30 minutes, and the whole cell lysate was analyzed by western blotting. (C, D) Spontaneous production of IFN-I by peritoneal cells or BM-derived mast cells. The IFN-I expression was determined by qRT-PCR. *n* = 3 for each group. **P < 0.01; *P < 0.05; n.s., not significant. Original images in B can be found in S2 Data. Underlying data in C and D can be found in S1 Data. BM, bone marrow; BMMC, bone marrow–derived mast cell; IFNAR, Interferon-α/β receptor; IFN-I, type I interferon; IgE, immunoglobulin E; pSTAT1, phospho-STAT1; qRT-PCR, quantitative reverse transcription PCR; RQ, relative quantity; STAT, signal transduction and activator of transcription; WT, wild-type.

Constitutive IFN-I expression and secretion in the steady state are known to be important for maintaining the homeostasis of various types of cells and tissues [31]. The increased systemic and local anaphylaxis in *Ifnar1*^*−/−*^ mice strongly suggested that IFN-I in the steady state directly or indirectly influences mast cells. We therefore examined whether the properties of mast cells in peripheral niches were under the control of IFN-I. WT mast cells expressed IFNAR1 on their surface, although the expression level was low (Fig 2A). In contrast, the peak shift of IFNAR1 staining detected in WT mast cells was completely abolished in *Ifnar1*^*−/−*^ mast cells (Fig 2A). The IFNAR1 expressed on mast cells was functional, given that exogenously added IFN-I induced Stat1 tyrosine phosphorylation in WT but not in *Ifnar1*^*−/−*^ mast cells (Fig 2B). This observation supported previous reports that IFN-I affects mast cell functions [21].

We next examined whether mast cells have access to IFN-I in the steady state. The transcriptions of a panel of IFN-I genes were detectable in ex vivo peritoneal cells and in fibroblasts used for a preparation of in vitro–differentiated mast cells (connective tissue–type mast cells; CTMCs) (Fig 2C and S1 Fig). These results suggested that mast cells could be exposed to IFN-I in their environment. We also found that mast cells themselves transcribed IFN-I mRNA (Fig 2D). However, IFN-I proteins were not detectable in CTMC culture supernatants in ELISA. Although we could not detect IFN-I secretion from cultured mast cells, we speculated that mast cells receive and respond to IFN-I continuously in their niche in the steady state, and the loss of IFN-I signaling in the *Ifnar1*^*−/−*^ mice altered the nature of the mast cells, which may have caused the exacerbated anaphylactic reaction.

### Blockade of IFN-I signaling increases the severity of IgE-mediated systemic anaphylaxis

**Fig 3:**
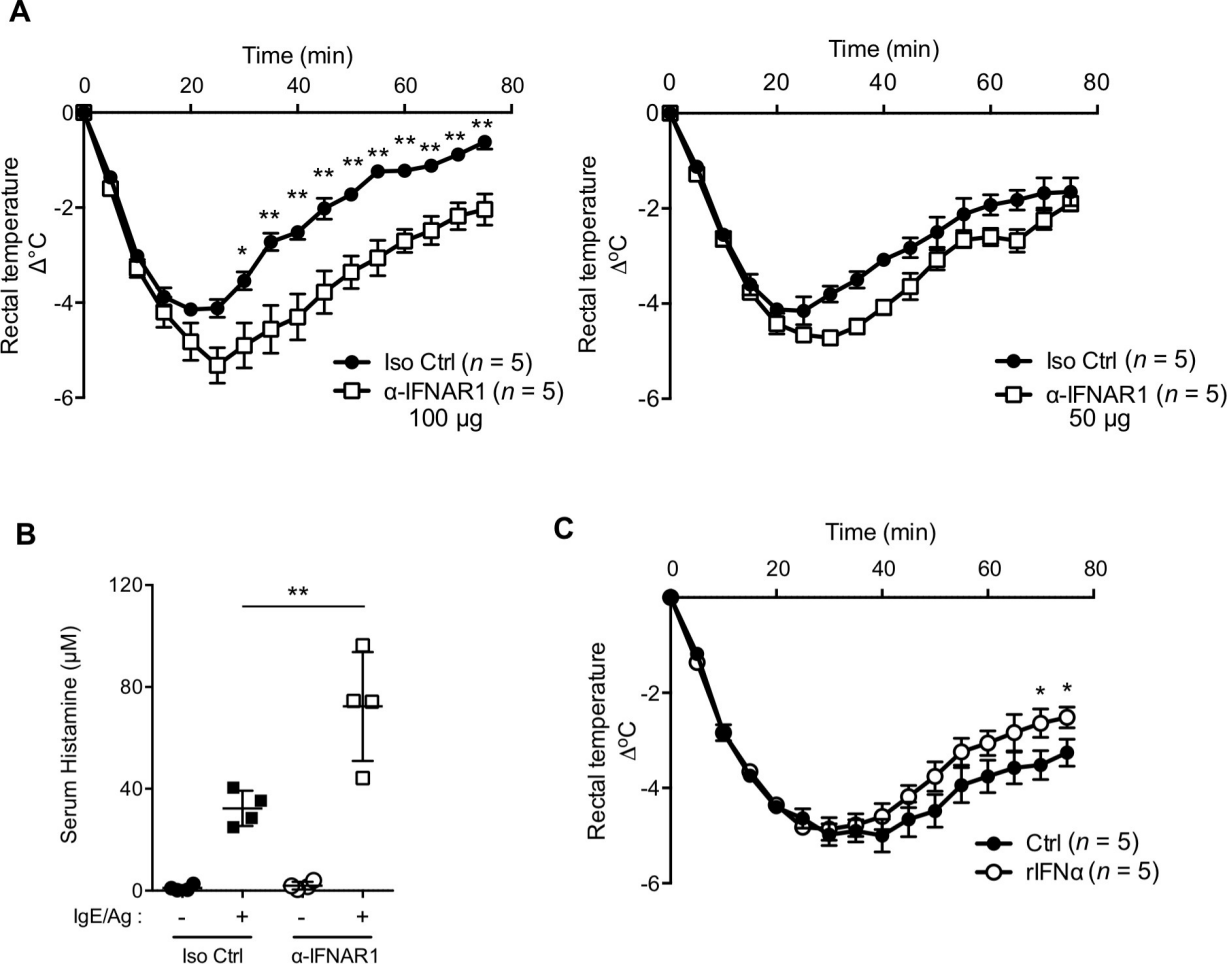
Modulation of IFN-I signaling altered the systemic anaphylaxis. (A) WT mice treated with an anti-IFNAR1 mAb that blocks type I IFN-I binding (*n* = 5) or isotype control mouse IgG1 (*n* = 5) on days −2 and −1 (left, 100 μg/administration; right, 50 μg/administration) and sensitized with IgE on day −1 were challenged with antigen on day 0. Hypothermia was monitored for 75 minutes. **P < 0.01 Results are shown as the mean ± SEM of values representative of three independent experiments. (B) Serum histamine level upon IgE-mediated passive systemic anaphylaxis in mice pretreated with either the anti-IFNAR1 antibody or isotype control, as in the left panel of (A), was determined by competitive enzyme immunoassay (EIA). *n* = 4 for each group. **P < 0.01 as determined by one-way ANOVA with Tukey’s test. Results are shown as the mean ± SD of values representative of three independent experiments. (C) IgE-mediated passive systemic anaphylaxis in mice pretreated with IFN-α (*n* = 5 for each group). *P < 0.05 (A, C) Results are shown as the mean ± SEM of values representative of three independent experiments. Underlying data from A to C can be found in S1 Data. Ctrl, control; IFNAR1, interferon-α/β receptor subunit 1; IFN-I, type I interferon; IgG1, immunoglobulin G1; IgE, immunoglobulin E; Iso, isotype control; mAb, monoclonal antibody; rIFNα, recombinant interferon-α; WT, wild-type.

To examine the above possibility, we investigated the effect of an anti-IFNAR1 antibody that blocks the binding of the IFN-I that is present in the mast cell niche in the steady state. When WT mice were treated with the anti-IFNAR1 antibody, they exhibited more severe hypothermia in association with IgE-mediated systemic anaphylaxis compared with control mice, and the severity of the hypothermia increased as greater amounts of anti-IFNAR1 antibodies were injected (Fig 3A). Importantly, the anti-IFNAR1 antibody treatment also increased the histamine released into the serum of WT mice after challenging with IgE and antigen (Fig 3B). These data collectively indicated that continuous signaling through IFNAR1 limits histamine secretion and systemic anaphylaxis. We also observed that the exogenous administration of IFN-I slightly but significantly ameliorated the systemic anaphylaxis-associated hypothermia (Fig 3C). This observation was consistent with previous reports showing a suppressive effect of IFN-I on mast cell histamine secretion. This result also suggested that, although mast cells in the steady state are under the control of endogenously produced IFN-I in their niches, exogenously added IFN-I could provide an additional effect to limit mast cell functions.

### Loss of IFN-I signaling causes an increase in secretory granule formation

**Fig 4:**
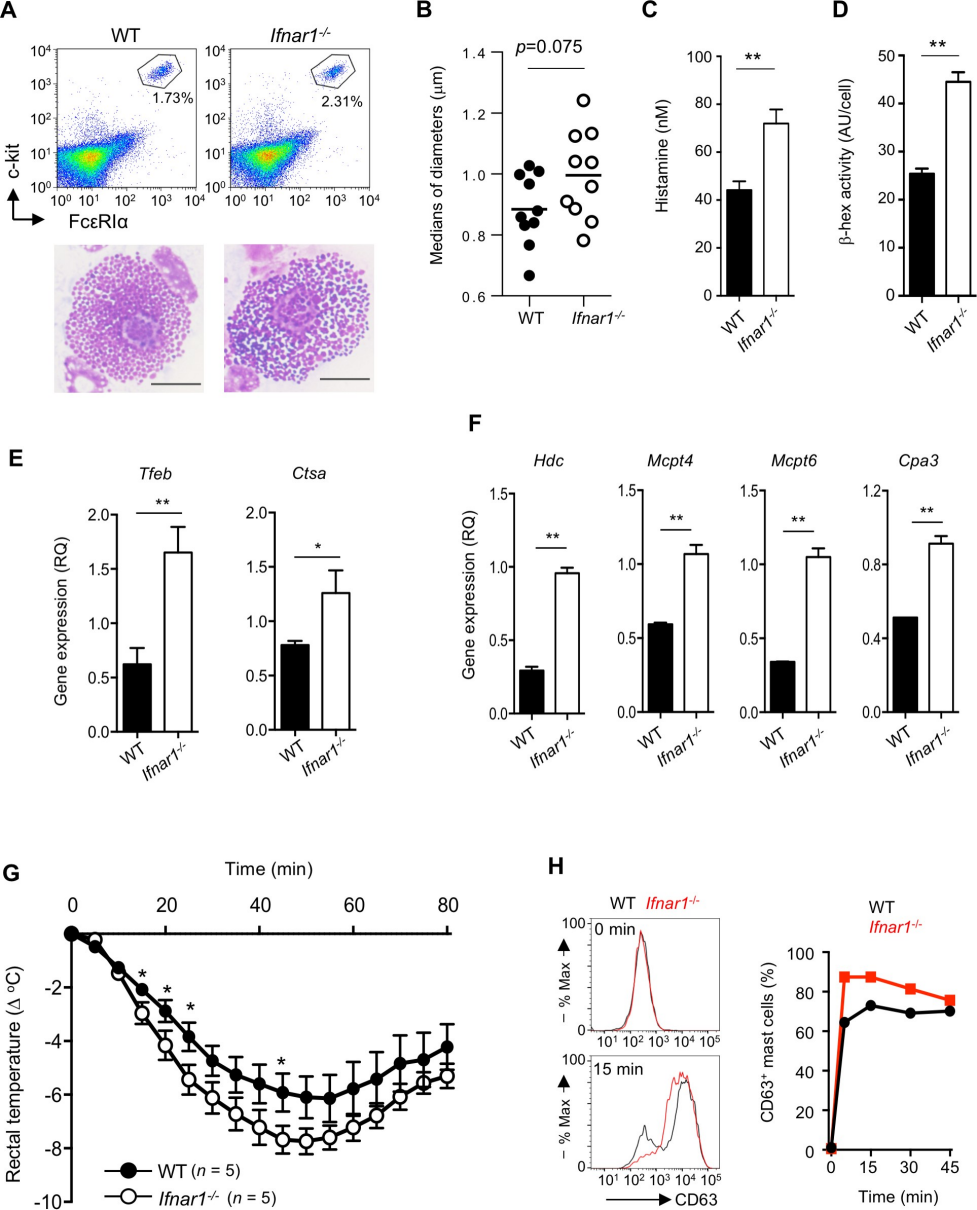
Loss of type I IFN signaling enhanced the terminal maturation of mast cells. (A) Morphology of the peritoneal mast cells in WT and *Ifnar1*^−/−^ mice. Peritoneal mast cells sorted as shown in the dot plots were stained with Diff-Quik and visualized by microscopy. Bars represent 5 μm. Representative images from three independent experiments are shown. (B) Purified peritoneal mast cells were analyzed by electron microscope as shown in S2 Fig, and the granule diameters of single WT or *Ifnar1*^*−/−*^ peritoneal mast cells were measured by Image J (ver 1.51m9). Dots indicate individual cells, and bars indicate mean values (*n* = 10, each group). (C, D) Development of the intracellular granules in peritoneal mast cells. The intracellular histamine level (C) or intracellular β-hexosaminidase activity (D) in WT or *Ifnar1*^−/−^ mast cells was quantified. **P < 0.01. Results are representative of three independent experiments. (E, F) Gene expression in WT or *Ifnar1*^−/−^ CTMCs was analyzed by RT-qPCR. *Hprt* was used as an internal control. **P < 0.01, *P < 0.05. (G) Compound 48/80–induced hypothermia in WT and *Ifnar1*^−/−^ mice (*n* = 5 for each group). *P < 0.05 as determined by one-tailed *t* test. (H) CD63 exposure on the cell surface upon FcεRI ligation in WT or *Ifnar1*^−/−^ CTMCs analyzed by flow cytometry. Histogram (left) or proportion of CD63^+^ cells at the indicated time points (right). Results are shown as the mean ± SD (B-F) or the mean ± SEM (G) of values representative of three independent experiments. Underlying data from B to H can be found in S1 Data. CTMC, connective tissue–type mast cell; *Ctsa*, cathepsin A; FcεRI, high affinity immunoglobulin E receptor; *Hprt*, hypoxanthine guanine phosphoribosyl transferase; IFN, interferon; RT-qPCR, quantitative reverse transcription PCR; *Tfeb*, transcription factor EB; WT, wild-type.

We next analyzed the characteristics of the WT and *Ifnar1*^*−/−*^ mast cells to determine the action point of the IFN-I. The expression levels of FcεRI and the cellular homolog of the transforming gene of a feline retrovirus (v-Kit) (c-kit) on the peritoneal mast cells in these mice were comparable (Fig 4A). However, the microscopic appearance of the secretory granules was different in the *Ifnar1*^*−/−*^ compared with the WT peritoneal mast cells. While the secretory granules in the WT mast cells were homogenous and of uniform size, those in the *Ifnar1*^*−/−*^ mast cells were of variable size and stainability, and included large granules (Fig 4A). Transmission electron microscopic analyses also showed the enlarged tendency of granule diameters in *Ifnar1*^*−/−*^ CTMC compared with WT CTMC (Fig 4B and S2 Fig). Importantly, the *Ifnar1*^*−/−*^ peritoneal mast cells stored significantly more histamine compared to the WT peritoneal mast cells (Fig 4C). In addition, the amount of stored β-hexosaminidase, a lysosomal enzyme, was also higher in the *Ifnar1*^*−/−*^ peritoneal mast cells (Fig 4D). Similar results were obtained when we analyzed CTMCs (S3 Fig). These results strongly suggested that the secretory granule synthesis was increased by the loss of IFNAR1. This notion was supported by the finding that the expression of *Tfeb*, which is the master regulator of lysosome/LRO biogenesis, was increased in the *Ifnar1*^*−/−*^ CTMCs (Fig 4E). Accordingly, the expression of the cathepsin A (*Ctsa*) gene, a TFEB target gene, was also up-regulated in the *Ifnar1*^*−/−*^ CTMCs (Fig 4E). The elevated secretory granule formation in the absence of IFN-I signaling was confirmed by increased granule-associated gene expressions, including that of histidine decarboxylase and secretory granule proteases in the *Ifnar1*^*−/−*^ CTMCs (Fig 4F). Consistent with these observations, the FcεRI-independent systemic anaphylaxis, in which compound 48/80 administration triggers the granule exocytosis of mast cells in the absence of IgE and antigen, was more severe in *Ifnar*^*−/−*^ than in WT mice (Fig 4G). Taken together, these results indicated that IFN-I limits mast cell functions by restricting the secretory granule biogenesis.

The increased TFEB expression prompted us to compare the ability of LRO exocytosis between WT and *Ifnar1*^*−/−*^ CTMCs by evaluating the CD63 surface expression, because TFEB promotes lysosome fusion with the plasma membrane. A higher CD63 surface expression was detected in the *Ifnar1*^*−/−*^ mast cells compared with the WT ones after FcεRI cross-linking (Fig 4H). This finding indicated that IFN-I also limits mast cell secretory granule exocytosis. Based on these observations, it appeared that increases in both the secretory granule formation and the granule exocytosis in *Ifnar1*^*−/−*^ mast cells contributed to the exacerbation of systemic anaphylaxis and the elevated histamine in the sera.

### IFNAR1 loss exerts slight effects on FcεRI-proximal signaling events

**Fig 5:**
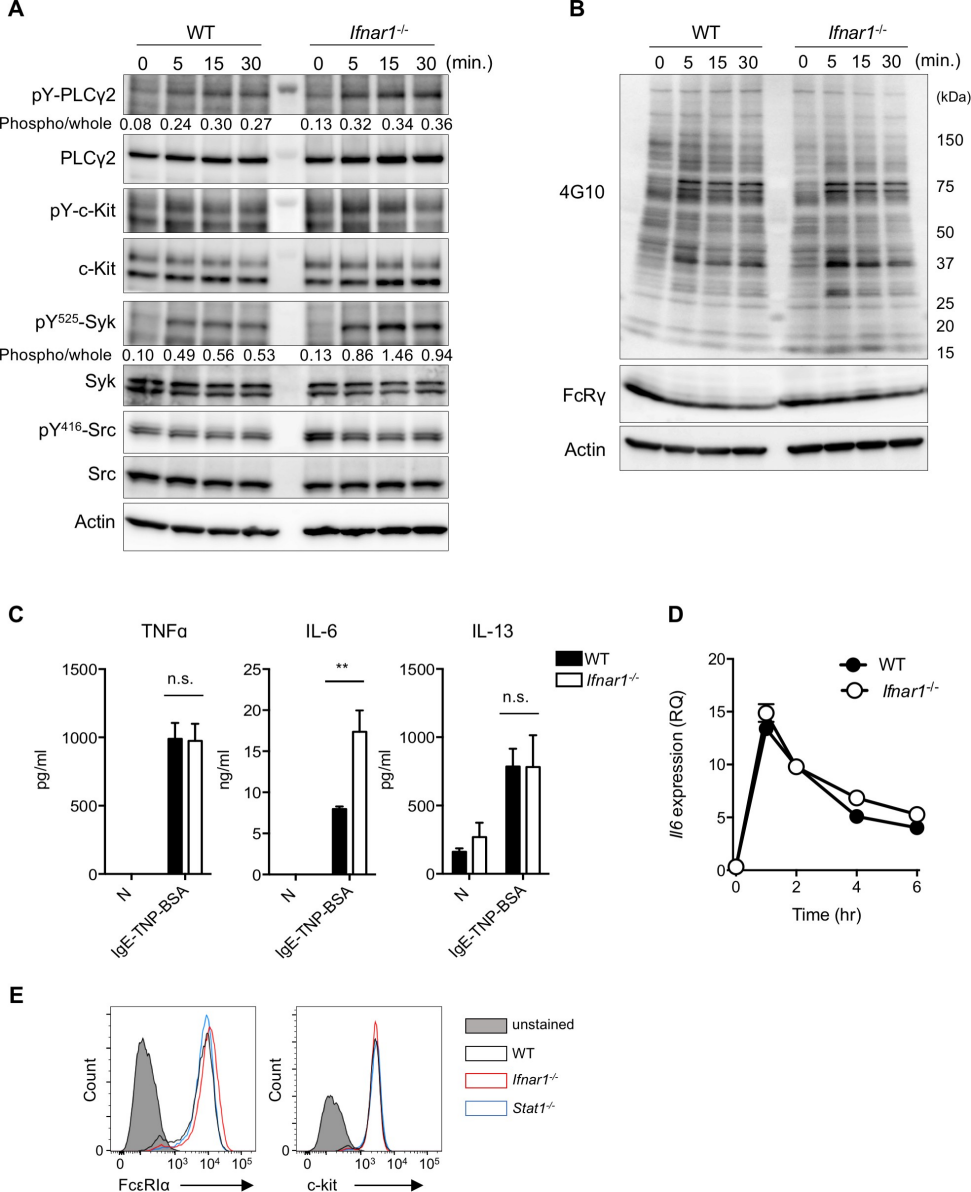
Type I IFN affected FcεRI-mediated phosphorylation events and posttranslational regulation of IL-6 production. (A, B) FcεRI-mediated signaling upon its ligation in CTMCs was analyzed by western blotting. WT or *Ifnar1*^−/−^ CTMCs sensitized with IgE were stimulated with TNP_4_-BSA for the indicated periods. Results shown are representative of three independent experiments. (C) Cytokine production upon FceRI ligation in CTMCs. The TNFα, IL-6, or IL-13 level in the culture supernatant was determined by ELISA. **P < 0.01; n.s., not significant. Results are shown as the mean ± SD. (D) *Il6* gene expression after FcεRI ligation in CTMCs, quantified by qRT-PCR. Each point represents the mean value from triplicates. (E) Expression level of FcεRiα or c-kit on the cell surface of WT, *Ifnar1*^−/−^, or *Stat1*^−/−^ CTMCs, analyzed by flow cytometry. Results shown are representative of three independent experiments. Original images in A and B can be found in S2 Data. Underlying data in C and D can be found in S1 Data. BSA, bovine serum albumin; CTMC, connective tissue–type mast cell; FcεRI, high affinity immunoglobulin E receptor; IFN, interferon; IgE, immunoglobulin E; IL-6, interleukin-6; pY-PLCγ2, tyrosine-phosphorylated phospholipase Cγ2; qRT-PCR, quantitative reverse transcription PCR; RQ, relative quantity; Src, Rous sarcoma oncogene; Syk, spleen tyrosine kinase; TNFα, tumor necrosis factor α; TNP, trinitrophenyl; WT, wild-type.

We further examined whether IFNAR1 loss altered the FcεRI-mediated mast cell responses. For this analysis, we compared biochemical FcεRI-proximal signaling events between WT- and *Ifnar1*^*−/−*^-derived CTMCs. We detected an increase in spleen tyrosine kinase (Syk) phosphorylation in the *Ifnar1*^*−/−*^ CTMCs but little difference in other FcεRI-proximal signaling molecules such as phospholipase Cγ2 (PLCγ2) (Fig 5A). The pattern of FcεRI-dependent protein tyrosine phosphorylation in total cell lysates was also slightly affected by the IFNAR1 deficiency (Fig 5B). While the FcεRI-dependent productions of tumor necrosis factor α (TNFα) and interleukin (IL)-13 were not affected by the absence of IFNAR1 (Fig 5C), the FcεRI-mediated IL-6 secretion was enhanced in the *Ifnar1*^*−/−*^ versus the WT CTMCs (Fig 5C). However, the IL-6 gene transcription was comparable between the WT and *Ifnar1*^*−/−*^ CTMCs (Fig 5D), indicating that the increased FcεRI-triggered IL-6 secretion in the absence of IFN-I signaling was not due to a transcriptional alteration but probably caused by the alteration of posttranscriptional regulations. This finding also suggested that the FcεRI-mediated signaling cascades related to these cytokine gene expressions were not affected by the absence or presence of IFNAR1-mediated signaling. In our hands, the number of CTMCs collected and the expression levels of surface molecules such as FcεRI and c-Kit were not significantly different between the *Ifnar1*^*−/−*^ and WT CTMCs (Fig 5E). Collectively, our results suggested that IFN-I in the steady state also limits FcεRI-dependent Syk activation but does not play an essential part of cytokine productions by mast cells.

### IFN-I limits the terminal maturation of mast cells

**Fig 6:**
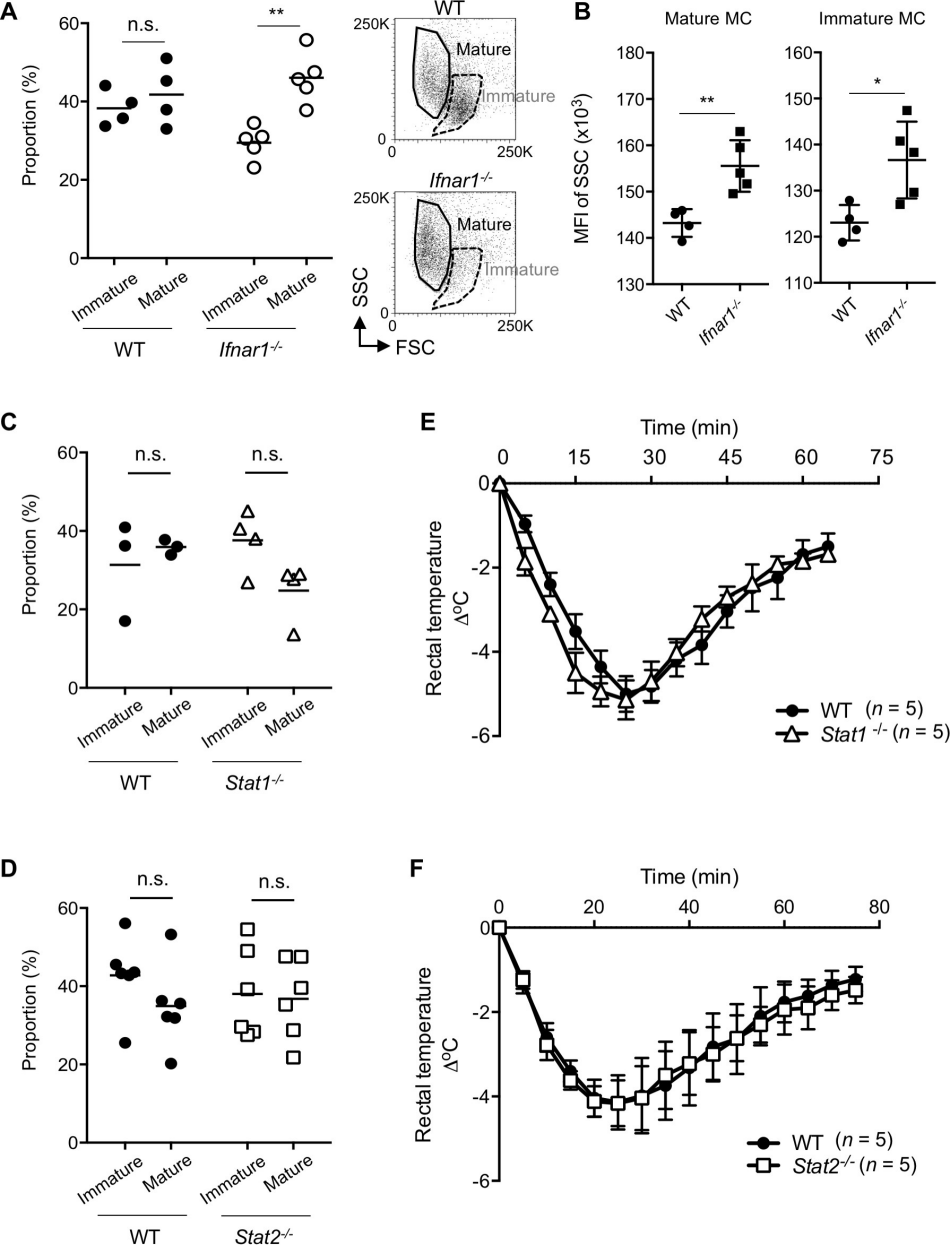
IFN-I signaling suppressed mast cell maturation in a Stat1- or Stat2-independent manner. (A) Immature or mature mast cells in the peritoneum of naïve WT or *Ifnar1*^−/−^ mice was analyzed by flow cytometry; the FcεRI^+^, c-kit^+^ mast cells were further divided into two groups (immature and mature), and their proportions were calculated. Left, each circle represents the value from one mouse; horizontal bars show the mean values. **P < 0.01; n.s., not significant. (B) MFI of the SSC of mature or immature mast cells as distinguished in (A) was determined by flow cytometry. Each symbol represents the value from one mouse; horizontal bars and error bars show the mean and SD. **P < 0.01, * *P* < 0.05. (C, D) Immature and mature mast cells in the peritoneum of naïve mice were analyzed by flow cytometry; WT versus *Stat1*^−/−^ mice (C), and WT versus *Stat2*^−/−^ mice (D). Each symbol represents the value from one mouse; horizontal bars show the mean values. n.s., not significant. (E, F) IgE-mediated passive systemic anaphylaxis. WT and *Stat1*^−/−^ mice (E) or WT and *Stat2*^−/−^ mice (F) were sensitized with anti-TNP IgE followed by challenge with TNP_4_-BSA, and the body temperature was monitored for the indicated periods. *n* = 5 for each group. There were no statistically significant differences. Results are shown as the mean ± SEM of values representative of three independent experiments. Underlying data from A to F can be found in S1 Data. BSA, bovine serum albumin; c-kit, cellular homolog of the transforming gene of a feline retrovirus (v-Kit); FcεRI, high affinity immunoglobulin E receptor; IFN-I, type I interferon; IgE, immunoglobulin E; MC, mast cell; MFI, mean fluorescence intensity; SSC, side scatter; Stat, signal transduction and activator of transcription; TNP, trinitrophenyl; WT, wild-type.

The granule synthesis in mast cells is known to be correlated with their maturation process. Mast cell maturity can be evaluated by flow cytometry, by which peritoneal mast cells can be classified into two different populations, immature and mature mast cells [32]. Mature mast cells also possess a high side scatter (SSC) and low forward scatter (FSC) light profile, which probably reflects the mast cell granule state. Therefore, we examined whether a difference in SSC was detectable in the WT versus *Ifnar1*^*−/−*^ mast cells. Flow cytometric analyses of the peritoneal mast cells revealed that the ratio of mature mast cells to total peritoneal mast cells was significantly higher in *Ifnar1*^*−/−*^ mice than in WT mice and that the proportion of immature mast cells was conversely lower in the *Ifnar1*^*−/−*^ mice than in WT mice (Fig 6A). In addition, significantly higher SSC mean fluorescence intensity (MFI) was detected in both mature and immature *Ifnar1*^*−/−*^ mast cells than in WT mast cells (Fig 6B). These data were consistent with the results shown in Fig 4. Peritoneal mast cell numbers and proliferation ability of *Ifnar*^*−/−*^ BMMC/CTMC were not significantly different between WT and *Ifnar*^*−/−*^ mice (Fig 1E). Furthermore, as far as we assessed peritoneal mast cell proliferation using ethynyl-deoxyuridine (EdU), we could not detect the proliferative fractions in peritoneal mast cells. Collectively, it is plausible that the increase of mature mast cell proportion in the *Ifnar1*^*−/−*^ peritoneal cavity is primarily caused by IFN-I’s effect on mast cell maturation rather than on proliferation, and the absence of IFN-I signaling in mast cells promotes their maturation.

### Stat1 and Stat2 are not sufficient to elicit IFN-I’s effect on mast cell homeostasis

Both STAT1 and STAT2 play a central role in IFN-I–dependent signaling [6], and therefore Stat1 or Stat2 deficiency should phenocopy the IFNAR1 deficiency in various IFN-I–dependent cellular responses. Unexpectedly, however, both *Stat1*^*−/−*^ and *Stat2*^*−/−*^ mice exhibited comparable frequencies of mature and immature peritoneal mast cells to those of WT mice and did not show a progression of mast cell maturation (Fig 6C and 6D). Furthermore, the severity of hypothermia in the FcεRI-mediated systemic anaphylaxis in *Stat1*^*−/−*^ or *Stat2*^*−/−*^ mice was comparable to that in WT mice (Fig 6E and 6F). These results clearly indicated that Stat1 and Stat2 were not sufficient to mediate IFN-I’s functions in mast cells.

### Stat3 is required for IFN-I’s negative regulation of mast cell functions

**Fig 7:**
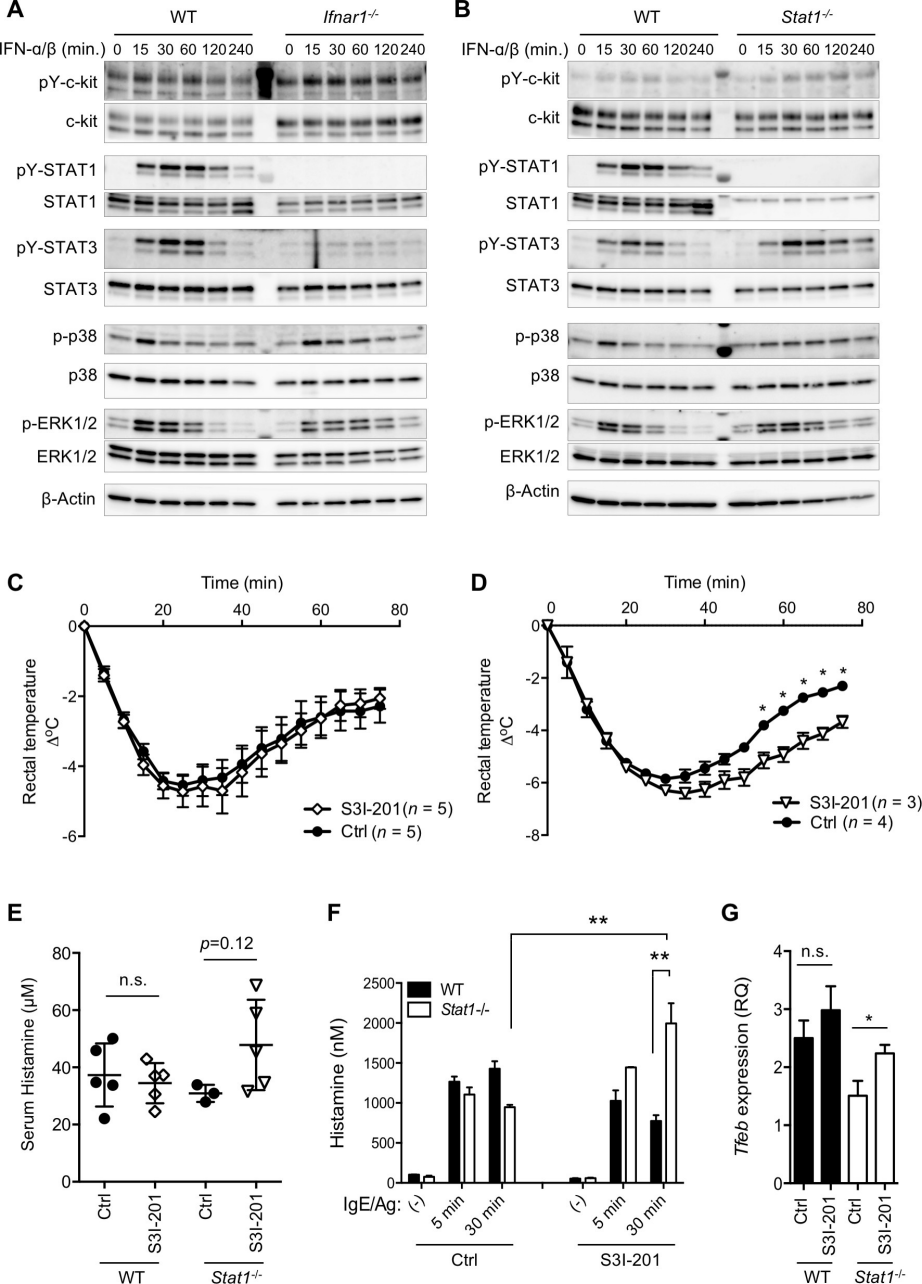
IFN-I–dependent Stat3 activation contributes to the augmentation of secretory granule formation and systemic anaphylaxis. (A, B) Type I IFN signaling in BMMCs. WT or *Ifnar1*^−/−^ (A), and WT or *Stat1*^−/−^ (B) mast cells were treated with IFN-α/β for the indicated periods, and the whole-cell lysate was analyzed by western blotting. Results are representative of three independent experiments. (C) WT mice treated with the Stat3 inhibitor S3I-201 (*n* = 5) or vehicle (*n* = 5) on day −1 and 2 hours before antigen challenge on day 0 (100 μg/administration), and sensitized with IgE on day −1 were challenged with antigen on day 0. Hypothermia was monitored for 75 minutes. Two groups were not significantly different over all time points. (D) *Stat1*^−/−^ mice were treated with S3I-201 (*n* = 3) or vehicle (*n* = 4), subjected to antigen challenge, and monitored for hypothermia as described for panel C. **P < 0.01, *P < 0.05. (E) Serum histamine level upon IgE-mediated passive systemic anaphylaxis in mice pretreated with either S3I-201 or vehicle, as in the left panel of (A), was determined by competitive EIA. Each symbol represents the value from one mouse; horizontal bars and error bars show the mean and SD. Statistical significance was determined by one-way ANOVA with Tukey’s test. Results are representative of three independent experiments. (F) Histamine release upon FcεRI ligation from WT or *Stat1*^−/−^ CTMCs treated with S3I-201 was determined by EIA. **P < 0.01, by one-way ANOVA with Tukey’s test. Results are shown as the mean ± SD of values representative of three independent experiments. (G) *Tfeb* expression in WT or *Stat1*^−/−^ CTMCs treated with S3I-201 was analyzed by RT-qPCR. *P < 0.05; n.s., not significant. Results are shown as the mean ± SD of values determined from three independent experiments. Original images in A and B can be found in S3 Data. Underlying data from C to G can be found in S1 Data. BMMC, bone marrow–derived mast cell; c-kit, cellular homolog of the transforming gene of a feline retrovirus (v-Kit); CTMC, connective tissue–type mast cell; Ctrl, control; EIA, enzyme immunoassay; ERK, Extracellular signal-regulated kinase; FcεRI, high affinity immunoglobulin E receptor; IFN-I, type I interferon; IgE, immunoglobulin E; pY, phosphotyrosine; RQ, relative quantity; RT-qPCR, quantitative reverse transcription PCR; Stat, signal transduction and activator of transcription; *Tfeb*, transcription factor EB; WT, wild-type.

Previous studies demonstrated that IFN-I’s binding to IFNAR1 induces STAT3 phosphorylation [11–13], although the significance of this effect was unclear. As expected, the Stat3 in WT mast cells was tyrosine-phosphorylated by IFN-I treatment (Fig 7A). Neither Stat1 nor Stat3 was phosphorylated by the same treatment in *Ifnar1*^*−/−*^ CTMCs (Fig 7A), indicating that the phosphorylation of these two STATs was indeed IFNAR-dependent. Stat3’s tyrosine phosphorylation was still detectable in *Stat1*^*−/−*^ CTMCs and appeared to increase (Fig 7B), consistent with a previous study [33]. Therefore, we tested Stat3’s contribution to the IFN-I–mediated mast cell regulation using a STAT3 inhibitor, S3I-201. WT mice treated with S3I-201 showed a similar degree of hypothermia as control-treated WT mice (Fig 7C). Interestingly, however, when *Stat1*^*−/−*^ mice were treated with the STAT3 inhibitor, the IgE-mediated systemic anaphylaxis was exacerbated, although the severity of the hypothermia in S3I-201–treated *Stat1*^*−/−*^ mice appeared to be milder than that of the *Ifnar*^*−/−*^ mice (Fig 7D). Consistent with this observation, S3I-201–treated *Stat1*^*−/−*^ but not WT mice tended to have a higher serum histamine concentration (Fig 7E). In addition, *Stat1*^*−/−*^ CTMCs secreted significantly higher amounts of histamine than WT CTMCs after S3I-201 treatment (Fig 7F). Importantly, the S3I-301–treated *Stat1*^*−/−*^ CTMCs also showed increased *Tfeb* transcription compared with vehicle-treated *Stat1*^*−/−*^ CTMCs (Fig 7G). These results strongly suggested that Stat1 and Stat3 act redundantly downstream of IFNAR and that the loss of both STAT molecules releases mast cells from the IFN-I–mediated limitations on secretory granule biogenesis and functional maturation.

## Discussion

We here investigated the effect of IFN-I on mast cell functions using *Ifnar1*^*−/−*^ mice, and revealed that IFN-I has a critical role in mast cell homeostasis to limit mast cell–dependent anaphylaxis. IFNAR1 deficiency caused an exacerbated systemic anaphylaxis that was associated with increased histamine in the circulation. This outcome was primarily due to the increased storage and exocytosis of secretory granule components, including histamine, in the mast cells. This scenario was supported by the finding that compound 48/80 also induced a more severe systemic anaphylaxis in *Ifnar1*^*−/−*^ than in WT mice, which is known to be a mast cell–dependent but FcεRI-independent response [34]. We currently speculate that mast cells are continuously exposed to IFN-I in their peripheral environment because IFN-I genes were transcribed in mast cells and their niche cells in the steady state. This steady-state IFN-I exposure appears to confine mast cells to less mature states and to limit the onset of anaphylactic responses. Consistent with this scenario, the administration of an anti-IFNAR1 antibody that blocks IFN-I binding also exacerbated systemic anaphylaxis and increased the histamine level in the serum. Although several previous studies have demonstrated the suppressive effect of IFN-I treatment on the FcεRI-mediated histamine release by mast cells [21,22], our current study revealed a novel role of IFN-I in mast cell homeostasis, in particular, a significant contribution of the IFN-I signaling axis to LRO biogenesis.

In our experiments, the histamine levels in CTMC were appreciably lower than those in peritoneal mast cells. One possible reason for the difference in histamine levels between these two types of mast cells is that CTMC obtained after 4 days’ culture with IL-3/SCF plus feeder cells is still not fully matured. The observation that mature and immature mast cell subsets detected in peritoneal mast cells (Fig 6A) were not detectable in the CTMC population may support this notion. Furthermore, in electron microscopic analyses, we observed obvious differences in the electron density of secretory granules between peritoneal mast cells and CTMC; i.e., the granules in peritoneal mast cells showed high electron density, while those in CTMC showed low electron density. Although in vitro–differentiated CTMC seems to be less mature compared with peritoneal mast cells, the effect of IFN-I on mast cells could be detectable, and therefore it is conceivable that IFN-I has an effect on mast cells before they undergo full maturation.

Our current study also provides the novel and unexpected observations that STAT1 and STAT2 were insufficient to elicit the IFNAR1-mediated regulation of mast cell homeostasis. Instead, we revealed that Stat3 has an important role in the IFN-I–mediated regulation of mast cell homeostasis downstream of IFNAR1. Previous studies reported that in addition to Stat1 and Stat2, IFN-I induces Stat3 phosphorylation [11–13], although the significance of the Stat3 phosphorylation downstream of IFNAR1 was unclear. Our findings that *Stat1*^*−/−*^ mice showed normal systemic anaphylaxis and that the administration of a Stat3 inhibitor in a *Stat1*^*−/−*^ but not WT background caused the exacerbation of anaphylaxis strongly suggested that Stat1 and Stat3 function redundantly in this process. It is currently unclear whether Stat1/3’s nuclear translocation and transcriptional activity are required for IFN-I’s effect on mast cells.

It should be noted that the mast cell TFEB expression was increased both in the absence of IFN-I signaling and under Stat3 inhibition in the Stat1-deficient background. TFEB is a master regulator of lysosome biogenesis [25], and we have reported that this transcription factor regulates the secretory granule biogenesis in mast cells [30]. Therefore, it is possible that Stat1/3 mediate IFN-I’s effects in cooperation with TFEB, nonetheless, other transcription factors besides TFEB are possibly involved in this machinery. Although whether and how Stat3 and/or Stat1 participate in the regulation of TFEB expression are currently unclear, two recent studies demonstrating Stat3’s involvement in lysosome homeostasis may provide a clue for future experiments aimed at clarifying this mechanism [35,36]. One study showed that Stat3 associates directly with TFEB in the nucleus and partly suppresses TFEB’s function [35]. These observations are not inconsistent with our results, because a decreased Stat3 activation caused by the absence of IFN-I signaling could contribute to the restoration of TFEB activity. Another study reported that lysosomal stress induces Stat3 activation, which regulates lysosome hydrolase expression under the conditions of nutritional sufficiency [36]. This Stat3 function is TFEB-independent and was suggested to be one of the mechanisms for lysosome adaptation to lysosomal stress, such as oxidative stress. Thus, Stat3 could be involved in the regulation and adaptation of lysosomes. However, our current results showing that Stat3 apparently negatively regulates mast cell granule synthesis seem inconsistent with the positive regulatory role of Stat3 for lysosome regulation under stress. Because lysosome biogenesis and adaptation are probably regulated in a context-dependent manner, i.e., under fed or starved, or inflammatory or homeostatic conditions [25], it is conceivable that Stat3, TFEB, and other unknown molecules contribute differentially to these fundamental cellular responses in non-mutually exclusive manners.

A previous study showed that the Stat3 inhibitor C188-9, which blocks the phosphorylation of Stat3’s tyrosine 705 residue, efficiently ameliorates mast cell–mediated systemic anaphylaxis through a regulation of vascular permeability, based on a Stat3-transcriptional activity–dependent tight junction stabilization in endothelial cells [37]. These results are apparently inconsistent with our data obtained by treating WT mice with the Stat3 inhibitor S3I-201, in which we did not observe an amelioration of the systemic anaphylaxis. There are several possible reasons for the apparently different behavior of Stat3, such as C188-9’s potential inhibition of Stat1 and Stat5 in addition to Stat3, differences in the modes of action between these two Stat3 inhibitors, and different experimental conditions, including the period of Stat3 inhibitor administration. Although the effect of these two Stat3 inhibitors on systemic anaphylaxis appeared to be different, it is interesting that both C188-9 and S3I-201 caused an increase in histamine release in the circulation [37]. Thus, these two independent observations suggest that Stat3 is functionally linked to secretory granule homeostasis.

STAT3’s role in mast cells and allergic responses may be helpful for understanding the pathology of autosomal-dominant hyper-IgE syndrome (AD-HIES) [38–40]. AD-HIES patients carry a dominant-negative STAT3 mutation such as a missense mutation or in-frame deletion. These patients exhibit elevated serum IgE and frequently develop allergic or nonallergic inflammation. Food allergies and anaphylaxis are reported to be markedly diminished in AD-HIES patients with STAT3 mutations compared with other hyper-IgE patients with no STAT3 mutations [20]. In addition, transgenic mice expressing AD-HIES–associated *STAT3* mutations show an amelioration of mast cell–dependent systemic anaphylaxis [37]. It was also demonstrated that *STAT3* knock-down in a human mast cell line, LAD2, decreased the cells’ FcεRI-triggered degranulation [37]. Although hyper IgE may decrease the contribution of antigen-specific IgE to FcεRI cross-linking, these previous findings suggest that STAT3 positively regulates mast cell function, including degranulation, and support the idea that STAT3 inhibition is beneficial for controlling anaphylaxis [37]. It should be emphasized that our current observations do not contradict these previous findings, because all of the observations concerning AD-HIES reflect the loss of multiple STAT3-dependent signaling axes [41]. In contrast, our interpretation was based on data focusing on the STAT3 activity downstream of IFNAR1. Interestingly, the phosphorylation, nuclear translocation, and target gene expression of the AD-HIES–associated mutant STAT3 molecules are not different from those of WT STAT3 [40]. Therefore, how the mutant STAT3 behaves and causes AD-HIES–associated phenotypes is still largely unclear. Thus, even though there is abundant evidence that STAT3 contributes to mast cell functions and anaphylactic responses in the context of AD-HIES, we still do not fully understand its mechanism.

From a therapeutic point of view, even though mast cells in the steady state were under the control of IFN-I, it was meaningful that the exogenous administration of IFN-I could ameliorate the mast cell–dependent systemic anaphylaxis. Notably, this amelioration was achieved by a relatively short-term treatment with IFN-I, i.e., two pretreatments for two consecutive days before antigen challenge. In addition, the blockade of IFN-I signaling by an anti-IFNAR1 antibody in WT mice or STAT3 inhibition in the *Stat1*^*−/−*^ background significantly exacerbated the onset of systemic anaphylaxis and increased the serum histamine concentration when provided for only 2 days before sensitization. Taken together, it is plausible that IFN-I exerts a rapid effect on mast cells to alter the cells’ properties, including granule storage. Such a quick effect of IFN-I may be explained by the rapid mode of TFEB-mediated lysosome regulation [26]. However, the mechanisms of IFN-I action on systemic anaphylaxis need to be interpreted with caution; considering the broad targets of IFN-I in vivo, besides the suppression of LRO biogenesis in mast cells, other possible mechanisms in non-mast cells should be further investigated.

In conclusion, we demonstrated that IFN-I is important for maintaining mast cell homeostasis in the steady state. This study revealed a novel, homeostatic role of IFN-I in LRO biogenesis. Furthermore, we revealed that IFNAR1-mediated Stat3 activation is required for this function of IFN-I. Although our study raises many questions that still need to be answered, it also provides the promising possibility that targeting the machinery of LRO biogenesis or adaptation may represent a novel strategy for controlling mast cell functions.

## Materials and methods

### Ethics statement

All animal experiments were approved by the President of the National Center for Global Health and Medicine (NCGM) with the consideration of the institutional Animal Care and Use Committee (approval No. 18115) and were conducted in accordance with institutional procedures, national guidelines, and the relevant national laws on the protection of animals.

### Mice, cell lines, and reagents

*Ifnar1*-, *Stat1*-, and *Stat2*-deficient mice were obtained from The Jackson Laboratory (Bar Harbor, ME). C57BL/6J mice were purchased from CLEA Japan (Tokyo, Japan). The anti-TNP mouse IgE (IGELb4) was described previously [30]. TNP-conjugated BSA (LGC Biosearch Technologies, Middlesex, UK, T5050-10) and 4-methylumbellyferyl-N-acetyl-β-glucosaminide (4-MUAG) (Sigma, St. Louis, MO, M2133), recombinant mouse IL-3 and mouse SCF (Peprotech, Rockey Hill, NJ, 213–13 and 250–03, respectively), recombinant mouse Thrombopoietin (Wako Pure Chemicals, Osaka, Japan, 202–19611), recombinant mouse IFN-α (Miltenyi Biotec 130-093-131), and the STAT3 inhibitor S3I-201 (Merck Millipore, Burlington, MA; hereafter MM, 573102) were purchased.

### Mast cell preparation

BMMCs were prepared as follows. Bone marrow cells taken from the femur or tibia of WT, *Ifnar1*^−/−^, or *Stat1*^−/−^ mice were cultured with IL-3 for 6–8 weeks, and the purity of the BMMCs was confirmed by flow cytometry in which cells were stained with PE-anti-FcεRIα (MAR-1, Tonbo Biosciences, San Diego, CA, 50-5898-U100) and FITC-anti-c-kit (2B8, BioLegend, San Diego, CA, 105806) monoclonal antibodies (mAbs). For in vitro maturation, the BMMCs were cocultured with 3T3 fibroblasts in the presence of IL-3 and SCF [42]. Briefly, the BMMCs were cultured with BALB/c 3T3 fibroblasts in the presence of IL-3 and SCF for 4 days (with medium replacement on day 2), and the floating cells were collected and used as mature BMMCs (namely CTMCs). Peritoneal mast cells were enriched by the following procedure. Cells collected from the peritoneal lavage were stained with PE-anti-FcεRIα and FITC-anti-c-kit antibodies, and the double-positive cells were sorted on a FACSAria II (BD Biosciences, San Jose, CA) cell sorter.

### Systemic anaphylaxis model

For IgE-mediated passive systemic anaphylaxis, mice were sensitized with an intravenous (i.v.) injection of 10 μg anti-TNP IgE (IGELb4) and were challenged 24 hours later with 400 μg of TNP_4_-BSA. The rectal temperature of the mice was monitored for 75 minutes using a digital thermometer (Shibaura Electronics, Saitama, Japan, TD-320). Pharmacological modulation during the systemic anaphylaxis was performed as follows. To block IFNAR1, 50 or 100 μg of an anti-IFNAR1 antibody (MAR1-5A3, ThermoFisher, Waltham, MA, 16-5945-38) or IgG1 isotype control (ThermoFisher, 16-4714-85) was i.v. administered twice into WT mice, 2 days and 1 day before antigen challenge. These mice were sensitized with an anti-TNP IgE antibody 1 day before antigen challenge. For the treatment with IFN-α, 5,000 U of recombinant mouse IFN-α (Miltenyi Biotec, Bergisch Gladbach, Germany, 130-093-130) was intraperitoneally (i.p.) administered 2 days and 1 day before the antigen challenge, and the anti-TNP IgE antibody was i.v. injected 1 day before the antigen challenge. For Stat3 inhibition, the Stat3 inhibitor (S3I-201) was i.p. administered at 5 mg/kg 1 day and 2 hours before the antigen challenge.

To measure serum histamine levels, the mice were killed 90 seconds after the TNP_4_-BSA challenge, and the serum was immediately isolated from blood obtained by cardiac puncture. The histamine level in mast cell culture medium or serum was determined by a competitive enzyme immunoassay (EIA) (Bertin Pharma, Montigny le Bretonneux, France, #A05890). For the measurement of histamine levels in peritoneal mast cells, 6–8 × 10^4^ sorted mast cells were used, and in CTMC, 3 × 10^5^ CTMC were used in each experiment. For the compound 48/80–induced anaphylaxis model, compound 48/80 (Nacalai tesque, Kyoto, Japan, #09347–94) was i.p. administered into mice at 4 mg/kg, and the rectal temperature of the mice was monitored for 75 minutes using a digital thermometer.

### PCA model

For the PCA model, 100 ng of anti-TNP IgE was intradermally administered into the ear pinna using an Ito Microsyringe (Ito corporation, Japan, MS-N50), and the mouse was challenged 24 hours later with an i.v. injection of 200 μL of PBS containing 200 μg of TNP_4_-BSA and 0.5% Evans blue (Tokyo Chemical Industries, Tokyo, Japan, E0197), as an indicator of vascular leakage. Thirty minutes after the antigen challenge, the mice were euthanized, and the Evans blue dye was extracted by incubating the dissected ear in 700 μL of formamide (Nacalai tesque, 16228–05) at 63°C overnight. The absorbance of Evans blue in the extract was measured with a spectrophotometer at 620 nm.

### Degranulation assay and cytokine quantification

To measure degranulation upon FcεRI ligation, CTMCs were incubated overnight with anti-TNP IgE at 0.5 μg/mL (clone IGELb4) in medium containing IL-3. The cells were washed twice with prewarmed Tyrode’s buffer (10 mM HEPES [pH 7.4], 130 mM NaCl, 5 mM KCl, 1 mM MgCl_2_, 1.4 mM CaCl_2_, 5.6 mM D-glucose, and 0.1% BSA) and stimulated with TNP_4_-BSA in Tyrode’s buffer for the indicated periods. The culture supernatant was incubated with 4-MUAG (final concentration 0.5 mM) in citrate-phosphate buffer (pH 4.5; 52 mM citric acid, 94 mM Na_2_HPO_4_) for 15 minutes at 37°C, followed by the addition of Stop solution (2 M Na_2_CO_3_, 1.1 M glycine) to end the reaction. Fluorescence (excitation, 365 nm; emission, 450 nm) was measured on a microplate reader (VarioScan Flash, Thermo Fisher). The degranulation rate was calculated by dividing the β-hexosaminidase activity in the culture supernatant by that in the cell lysate, which was the supernatant of cells lysed with Tyrode’s buffer containing 0.5% Triton-X 100. Cytokine levels in the BMMC culture supernatants were measured using the Mouse ELISA Max Kit (BioLegend Mouse IL-6 ELISA MAX, 431302) or the Ready-Set-Go ELISA Kit (Thermo Fisher, 88-7324-88 for TNFα, 88-7137-88 for IL-13).

### Immunoblot analyses

The whole-cell lysate of BMMCs was subjected to SDS-PAGE, and the separated proteins were transferred to a membrane. The blots were then incubated with the target antibody, followed by the appropriate HRP-conjugated secondary antibody, and visualized by the LAS-3000 detection system (Fuji Photo Film, Japan). The following antibodies were used for immunoblot analyses: anti-Stat1 (Cell Signaling Technology; hereafter CST, #9172), anti-p-Stat1 (CST #7649), anti-Stat3 (CST #12640), anti-p-Stat3 (CST #9145), anti-PLCγ2 (Santa Cruz Biotechnology; hereafter SCB, sc-407), anti-p-PLCγ2 (CST #3871), anti-c-kit (SCB, sc-1494), anti-p-c-kit (CST #3319), anti-Syk (SCB, sc-929), anti-p-Syk (CST #2710), anti-Src (CST #2109), anti-p-Src family (CST #6943), anti-Phosphotyrosine (4G10)-HRP (MM 16–316), anti-FcεRI β chain (MM MABF38), anti-FcεRI γ chain (MM 06–727), anti-ERK1/2 (CST #4695), anti-p-ERK1/2 (CST #4370), anti-p38(CST #9212), anti-p-p38 (CST #9211), anti-β-Actin (CST #4970), and anti-α-Tubulin (CST #2125). To quantify the band intensities, Image Gauge (Fuji Photo Film, Tokyo, Japan) was used.

### Gene expression assays

The RNA from mast cells was extracted with Isogen (Nippon Gene, Japan, 311–02501) and reverse-transcribed using a SuperScript III cDNA Synthesis Kit (Thermo Fisher 11752050); otherwise, the CellAmp Direct Lysis and RT set (Takara, Japan, 3737S) was used. Quantitative RT-PCR was conducted using a StepOnePlus Real-Time PCR System (Thermo Fisher) with TaqMan probes for mouse *Il6*, *Ifna4*, *Ifna5*, *Ifnb1*, or *Hprt* (Thermo Fisher, Mm00446190_m1 for *Il6*, Mm00833969_s1 for *Ifna4*, Mm00833976_s1 for *Ifna5*, Mm00439552_s1 for *Ifnb1*, and Mm03024075_m1 for *Hprt*). The expression of *Hdc*, a gene involved in histamine synthesis, was quantified with the THUNDERBIRD SYBR qPCR mix (Toyobo, Japan, QPS-201) on a StepOnePlus Real-Time PCR System. Gene expressions were normalized to that of *Hprt*. The primers used in this study are listed in S1 Table.

### Flow cytometry

Single-cell suspensions were prepared from the peritoneal lavage, the spleen, or the bone marrow of mice, and the cells were stained with the indicated mAbs and analyzed by flow cytometry using a BD FACSVerse or BD FACSCalibur (BD Biosciences). The following antibodies were used for flow cytometry: PE- or APC-conjugated anti-FcεRI (Thermo Fisher 17-5898-82), Alexa Fluor647- (BioLegend 105818) or FITC-conjugated anti-c-kit (BioLegend 105806), and PE-anti-mouse IFNAR1 (BioLegend 127311). To detect the degranulation of mast cells after FcεRI ligation, APC-anti-mouse CD63 (Thermo Fisher 17-0631-80) was used. For the in vivo IgE-binding assay, 10 μg of the IGELb4 IgE antibody labeled with Alexa Fluor 647 using the Alexa Fluor 647 Microscale Protein Labeling Kit (Thermo Fisher A30009) was i.v. administered into mice, and 24 hours later, the mast cells in the peritoneal lavage were analyzed on a FACSVerse.

### Electron microscopy

Peritoneal MCs were separated as c-kit^+^, FcεRIα^+^ cells on FACSAria II and fixed in 2% glutaraldehyde in 0.1 M HEPES for overnight, attached on a poly-L-Lysine–coated plastic dish, postfixed with 1% OsO_4_ for 60 minutes at RT, followed by dehydration in ethanol, and finally embedded in epoxy resin. Sections were analyzed by electron microscope (JEM-1400; JEOL, Japan). Sizes of the intracellular granules were determined by measuring the length of the granules on TEM images using ImageJ software.

### Statistics

The statistical significance of differences in the mean ± SD of various groups was calculated with a two-tailed *t* test unless otherwise noted. For the comparison between more than two groups, Tukey’s test was applied as post hoc analyses in ANOVA. *P* values less than 0.05 were considered statistically significant. All animal data are presented as the mean ± SEM of at least 3 independent experiments.

## Supporting information

S1 Fig Expression of type I IFN genes from 3T3 fibroblasts or in vitro–differentiated mast cells (CTMCs). BALB/3T3 fibroblasts, WT, *Ifnar1*^−/−^, or *Stat1*^−/−^ CTMCs were subjected to qRT-PCR. Each point represents the value from individual sample; horizontal bars show the mean. Results are representative of three independent experiments. Underlying data can be found in S1 Data. BALB/3T3, immortalized BALB/c mice-derived fibroblast; CTMC, connective tissue–type mast cell; IFN, interferon; qRT-PCR, quantitative reverse transcription PCR; WT, wild-type.
(TIF)

S2 Fig Intracellular granules in peritoneal MCs. (A) Sorting strategy of peritoneal mast cells from WT or *Ifnar1*^−/−^ mice. Peritoneal MCs were separated as c-kit^+^, FcεRIα^+^ cells on FACSAria II. SSC of the separated mast cells was shown in the histogram. (B) Representative images of WT or *Ifnar1*^−/−^ peritoneal MCs were obtained by using transmission electron microscopy (TEM). Scale bars, 2 μm. (C) Diameter of the intracellular granules of WT or *Ifnar1*^−/−^ peritoneal MCs. Sizes of the intracellular granules were determined by measuring the length of the granules on TEM images using ImageJ software. (D-F) Statistical analysis of the character of intracellular granules. (D) Intracellular granule size of WT or *Ifnar1*^−/−^ peritoneal MCs. Dots indicate individual cells and bars indicate mean values (*n* = 10, each group). (E) The granule number in a single WT or *Ifnar1*^−/−^ peritoneal MC. Dots indicate individual cells and bars indicate mean values (*n* = 10, each group). *P < 0.05. (F) The granule number per the surface area of a single WT or *Ifnar1*^−/−^ peritoneal MC. Dots indicate individual cells and bars indicate mean values (*n* = 10, each group). Underlying data from C to F can be found in S1 Data. c-kit, cellular homolog of the transforming gene of a feline retrovirus (v-Kit); FcεRIα, high affinity immunoglobulin E receptor α subunit; MC, mast cell; SSC, side scatter; WT, wild-type.
(TIF)

S3 Fig Development of the intracellular granules in CTMCs. Intracellular histamine level (left) or intracellular β-hexosaminidase activity (right) in WT or *Ifnar1*^−/−^ mast cells was quantified. **P < 0.01, *P < 0.05, as determined by *t* test. Results are representative of three independent experiments. Underlying data can be found in S1 Data. CTMC, connective tissue–type mast cell; WT, wild-type.
(TIF)

S1 Table Primer sets used for qPCR in this study. qPCR, quantitative PCR.
(TIF)

S1 Data Underlying numerical data of all Figs. (XLSX)

S2 Data Original western blot images shown in Fig 2 and Fig 5. (PDF)

S3 Data Original western blot images shown in Fig 7. (PDF)

## Abbreviations

AD-HIES: autosomal-dominant hyper-IgE syndrome
BMMC: bone marrow–derived mast cell
BSA: bovine serum albumin
c-kit: cellular homolog of the transforming gene of a feline retrovirus (v-Kit)
CLEAR: coordinated lysosomal expression and regulation
CST: Cell Signaling Technology
CTMC: connective tissue–type mast cell
Ctsa: cathepsin A
EdU: ethynyl-deoxyuridine
EIA: enzyme immunoassay
FcεRI: high affinity immunoglobulin E receptor
FSC: forward scatter
IFNAR: interferon-α/β receptor, IFN-I, type I interferon
IgE: Immunoglobulin E
IL: interleukin
i.p.: intraperitoneally
ISG: IFN-stimulated gene
i.v.: intravenous
Jak: Janus family kinase
LRO: lysosome-related organelle
mAb: monoclonal antibody
MFI: mean fluorescence intensity
MM: Merck Millipore
mTORC1: mechanistic target of rapamycin complex 1
NCGM: National Center for Global Health and Medicine
PCA: passive cutaneous anaphylaxis
PLCγ2: phospholipase Cγ2
pSTAT1: phospho-STAT1
pY-PLCγ2: tyrosine-phosphorylated phospholipase Cγ2
qRT-PCR: quantitative reverse transcription PCR
RQ: relative quantity
SCB: Santa Cruz Biotechnology
SSC: side scatter
STAT: signal transduction and activator of transcription
Syk: spleen tyrosine kinase
TFEB: transcription factor EB
TNFα: tumor necrosis factor α
TNP: trinitrophenyl
Tyk2: tyrosine kinase 2
WT: wild-type
4-MUAG: 4-methylumbellyferyl-N-acetyl-β-glucosaminide

## References

[1] Gonzalez-Navajas JM, Lee J, David M, Raz E. Immunomodulatory functions of type I interferons. Nature reviews Immunology. 2012;12(2):125–35. Epub 2012/01/10. 10.1038/nri3133 22222875 PMC3727154

[2] Ng CT, Mendoza JL, Garcia KC, Oldstone MB. Alpha and Beta Type 1 Interferon Signaling: Passage for Diverse Biologic Outcomes. Cell. 2016;164(3):349–52. Epub 2016/01/30. 10.1016/j.cell.2015.12.027 26824652 PMC4733246

[3] Lopez de Padilla CM, Niewold TB. The type I interferons: Basic concepts and clinical relevance in immune-mediated inflammatory diseases. Gene. 2016;576(1 Pt 1):14–21. Epub 2015/09/28. 10.1016/j.gene.2015.09.058 26410416 PMC4666791

[4] Chill JH, Quadt SR, Levy R, Schreiber G, Anglister J. The human type I interferon receptor: NMR structure reveals the molecular basis of ligand binding. Structure. 2003;11(7):791–802. Epub 2003/07/05. 10.1016/s0969-2126(03)00120-5 . 12842042

[5] de Weerd NA, Vivian JP, Nguyen TK, Mangan NE, Gould JA, Braniff SJ, et al Structural basis of a unique interferon-beta signaling axis mediated via the receptor IFNAR1. Nature immunology. 2013;14(9):901–7. Epub 2013/07/23. 10.1038/ni.2667 . 23872679

[6] Platanias LC. Mechanisms of type-I- and type-II-interferon-mediated signalling. Nature reviews Immunology. 2005;5(5):375–86. Epub 2005/05/03. 10.1038/nri1604 . 15864272

[7] Stark GR, Darnell JE Jr. The JAK-STAT pathway at twenty. Immunity. 2012;36(4):503–14. Epub 2012/04/24. 10.1016/j.immuni.2012.03.013 22520844 PMC3909993

[8] Majoros A, Platanitis E, Kernbauer-Holzl E, Rosebrock F, Muller M, Decker T. Canonical and Non-Canonical Aspects of JAK-STAT Signaling: Lessons from Interferons for Cytokine Responses. Front Immunol. 2017;8:29 10.3389/fimmu.2017.00029 28184222 PMC5266721

[9] Mogensen TH. IRF and STAT Transcription Factors—From Basic Biology to Roles in Infection, Protective Immunity, and Primary Immunodeficiencies. Frontiers in immunology. 2018;9:3047 Epub 2019/01/24. 10.3389/fimmu.2018.03047 30671054 PMC6331453

[10] Ivashkiv LB, Donlin LT. Regulation of type I interferon responses. Nature reviews Immunology. 2014;14(1):36–49. Epub 2013/12/24. 10.1038/nri3581 24362405 PMC4084561

[11] Ho HH, Ivashkiv LB. Role of STAT3 in type I interferon responses. Negative regulation of STAT1-dependent inflammatory gene activation. The Journal of biological chemistry. 2006;281(20):14111–8. Epub 2006/03/31. 10.1074/jbc.M511797200 . 16571725

[12] Tsai MH, Lee CK. STAT3 Cooperates With Phospholipid Scramblase 2 to Suppress Type I Interferon Response. Frontiers in immunology. 2018;9:1886 Epub 2018/08/31. 10.3389/fimmu.2018.01886 30158934 PMC6104169

[13] Yang CH, Shi W, Basu L, Murti A, Constantinescu SN, Blatt L, et al Direct association of STAT3 with the IFNAR-1 chain of the human type I interferon receptor. The Journal of biological chemistry. 1996;271(14):8057–61. Epub 1996/04/05. 10.1074/jbc.271.14.8057 . 8626489

[14] Valent P, Akin C, Metcalfe DD. Mastocytosis: 2016 updated WHO classification and novel emerging treatment concepts. Blood. 2017;129(11):1420–7. Epub 2016/12/30. 10.1182/blood-2016-09-731893 28031180 PMC5356454

[15] Austen KF. Systemic mastocytosis. N Engl J Med. 1992;326(9):639–40. Epub 1992/02/27. 10.1056/NEJM199202273260912 . 1734257

[16] Kluin-Nelemans HC, Jansen JH, Breukelman H, Wolthers BG, Kluin PM, Kroon HM, et al Response to interferon alfa-2b in a patient with systemic mastocytosis. N Engl J Med. 1992;326(9):619–23. Epub 1992/02/27. 10.1056/NEJM199202273260907 . 1370856

[17] Pardanani A. Systemic mastocytosis in adults: 2017 update on diagnosis, risk stratification and management. Am J Hematol. 2016;91(11):1146–59. Epub 2016/10/21. 10.1002/ajh.24553 . 27762455

[18] Casassus P, Caillat-Vigneron N, Martin A, Simon J, Gallais V, Beaudry P, et al Treatment of adult systemic mastocytosis with interferon-alpha: results of a multicentre phase II trial on 20 patients. Br J Haematol. 2002;119(4):1090–7. Epub 2002/12/11. 10.1046/j.1365-2141.2002.03944.x . 12472593

[19] Lim KH, Pardanani A, Butterfield JH, Li CY, Tefferi A. Cytoreductive therapy in 108 adults with systemic mastocytosis: Outcome analysis and response prediction during treatment with interferon-alpha, hydroxyurea, imatinib mesylate or 2-chlorodeoxyadenosine. Am J Hematol. 2009;84(12):790–4. Epub 2009/11/06. 10.1002/ajh.21561 . 19890907

[20] Siegel AM, Stone KD, Cruse G, Lawrence MG, Olivera A, Jung MY, et al Diminished allergic disease in patients with STAT3 mutations reveals a role for STAT3 signaling in mast cell degranulation. J Allergy Clin Immunol. 2013;132(6):1388–96. Epub 2013/11/05. 10.1016/j.jaci.2013.08.045 24184145 PMC3881191

[21] Swieter M, Ghali WA, Rimmer C, Befus D. Interferon-alpha/beta inhibits IgE-dependent histamine release from rat mast cells. Immunology. 1989;66(4):606–10. Epub 1989/04/01. 2469645 PMC1385165

[22] Bissonnette EY, Chin B, Befus AD. Interferons differentially regulate histamine and TNF-alpha in rat intestinal mucosal mast cells. Immunology. 1995;86(1):12–7. Epub 1995/09/01. 7590871 PMC1383804

[23] Wernersson S, Pejler G. Mast cell secretory granules: armed for battle. Nature reviews Immunology. 2014;14(7):478–94. Epub 2014/06/07. 10.1038/nri3690 . 24903914

[24] Moon TC, Befus AD, Kulka M. Mast cell mediators: their differential release and the secretory pathways involved. Frontiers in immunology. 2014;5:569 Epub 2014/12/03. 10.3389/fimmu.2014.00569 25452755 PMC4231949

[25] Settembre C, Fraldi A, Medina DL, Ballabio A. Signals from the lysosome: a control centre for cellular clearance and energy metabolism. Nature reviews Molecular cell biology. 2013;14(5):283–96. Epub 2013/04/24. 10.1038/nrm3565 23609508 PMC4387238

[26] Settembre C, De Cegli R, Mansueto G, Saha PK, Vetrini F, Visvikis O, et al TFEB controls cellular lipid metabolism through a starvation-induced autoregulatory loop. Nature cell biology. 2013;15(6):647–58. Epub 2013/04/23. 10.1038/ncb2718 23604321 PMC3699877

[27] Sardiello M, Palmieri M, di Ronza A, Medina DL, Valenza M, Gennarino VA, et al A gene network regulating lysosomal biogenesis and function. Science. 2009;325(5939):473–7. Epub 2009/06/27. 10.1126/science.1174447 . 19556463

[28] Roczniak-Ferguson A, Petit CS, Froehlich F, Qian S, Ky J, Angarola B, et al The transcription factor TFEB links mTORC1 signaling to transcriptional control of lysosome homeostasis. Science signaling. 2012;5(228):ra42 Epub 2012/06/14. 10.1126/scisignal.2002790 22692423 PMC3437338

[29] Settembre C, Zoncu R, Medina DL, Vetrini F, Erdin S, Erdin S, et al A lysosome-to-nucleus signalling mechanism senses and regulates the lysosome via mTOR and TFEB. The EMBO journal. 2012;31(5):1095–108. Epub 2012/02/22. 10.1038/emboj.2012.32 22343943 PMC3298007

[30] Kobayashi T, Tsutsui H, Shimabukuro-Demoto S, Yoshida-Sugitani R, Karyu H, Furuyama-Tanaka K, et al Lysosome biogenesis regulated by the amino-acid transporter SLC15A4 is critical for functional integrity of mast cells. Int Immunol. 2017;29(12):551–66. Epub 2017/11/21. 10.1093/intimm/dxx063 29155995 PMC5890901

[31] Gough DJ, Messina NL, Clarke CJ, Johnstone RW, Levy DE. Constitutive type I interferon modulates homeostatic balance through tonic signaling. Immunity. 2012;36(2):166–74. Epub 2012/03/01. 10.1016/j.immuni.2012.01.011 22365663 PMC3294371

[32] Dahlin JS, Ding Z, Hallgren J. Distinguishing Mast Cell Progenitors from Mature Mast Cells in Mice. Stem Cells Dev. 2015;24(14):1703–11. Epub 2015/03/10. 10.1089/scd.2014.0553 25744159 PMC4499794

[33] Kim HS, Kim DC, Kim HM, Kwon HJ, Kwon SJ, Kang SJ, et al STAT1 deficiency redirects IFN signalling toward suppression of TLR response through a feedback activation of STAT3. Sci Rep. 2015;5:13414 Epub 2015/08/25. 10.1038/srep13414 26299368 PMC4547106

[34] McNeil BD, Pundir P, Meeker S, Han L, Undem BJ, Kulka M, et al Identification of a mast-cell-specific receptor crucial for pseudo-allergic drug reactions. Nature. 2015;519(7542):237–41. Epub 2014/12/18. 10.1038/nature14022 25517090 PMC4359082

[35] Li L, Sun B, Gao Y, Niu H, Yuan H, Lou H. STAT3 contributes to lysosomal-mediated cell death in a novel derivative of riccardin D-treated breast cancer cells in association with TFEB. Biochem Pharmacol. 2018;150:267–79. Epub 2018/02/25. 10.1016/j.bcp.2018.02.026 . 29476714

[36] Martinez-Fabregas J, Prescott A, van Kasteren S, Pedrioli DL, McLean I, Moles A, et al Lysosomal protease deficiency or substrate overload induces an oxidative-stress mediated STAT3-dependent pathway of lysosomal homeostasis. Nat Commun. 2018;9(1):5343 Epub 2018/12/19. 10.1038/s41467-018-07741-6 30559339 PMC6297226

[37] Hox V, O'Connell MP, Lyons JJ, Sackstein P, Dimaggio T, Jones N, et al Diminution of signal transducer and activator of transcription 3 signaling inhibits vascular permeability and anaphylaxis. J Allergy Clin Immunol. 2016;138(1):187–99. Epub 2016/03/08. 10.1016/j.jaci.2015.11.024 26948077 PMC4931983

[38] Mogensen TH. STAT3 and the Hyper-IgE syndrome: Clinical presentation, genetic origin, pathogenesis, novel findings and remaining uncertainties. JAKSTAT. 2013;2(2):e23435 Epub 2013/09/24. 10.4161/jkst.23435 24058807 PMC3710320

[39] Holland SM, DeLeo FR, Elloumi HZ, Hsu AP, Uzel G, Brodsky N, et al STAT3 mutations in the hyper-IgE syndrome. N Engl J Med. 2007;357(16):1608–19. Epub 2007/09/21. 10.1056/NEJMoa073687 . 17881745

[40] Minegishi Y, Saito M, Tsuchiya S, Tsuge I, Takada H, Hara T, et al Dominant-negative mutations in the DNA-binding domain of STAT3 cause hyper-IgE syndrome. Nature. 2007;448(7157):1058–62. Epub 2007/08/07. 10.1038/nature06096 . 17676033

[41] Sowerwine KJ, Holland SM, Freeman AF. Hyper-IgE syndrome update. Ann N Y Acad Sci. 2012;1250:25–32. Epub 2012/01/25. 10.1111/j.1749-6632.2011.06387.x 22268731 PMC4103910

[42] Ogasawara T, Murakami M, Suzuki-Nishimura T, Uchida MK, Kudo I. Mouse bone marrow-derived mast cells undergo exocytosis, prostanoid generation, and cytokine expression in response to G protein-activating polybasic compounds after coculture with fibroblasts in the presence of c-kit ligand. Journal of immunology. 1997;158(1):393–404. Epub 1997/01/01. . 8977215

