# Supplementary figures and images for: Type I interferon limits mast cell–mediated anaphylaxis by controlling secretory granule homeostasis
Source: PLoS Biol. 2019 Nov 15;17(11):e3000530. doi: 10.1371/journal.pbio.3000530 (PMC6892554; doi:10.1371/journal.pbio.3000530)

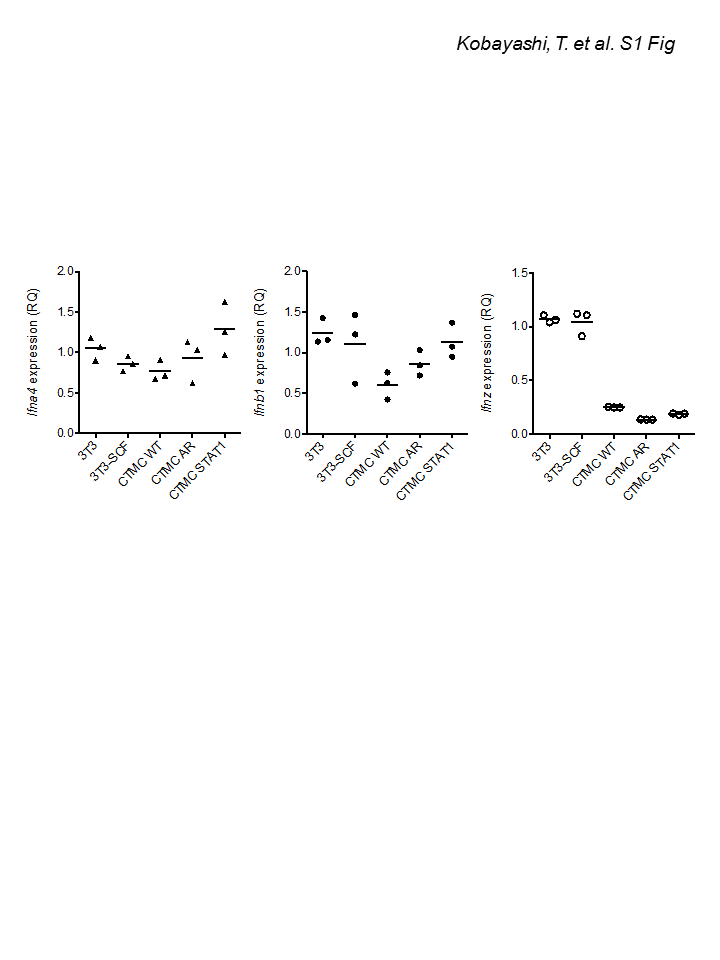

Supplement: S1 Fig — BALB/3T3 fibroblasts, WT, Ifnar1−/−, or Stat1−/− CTMCs were subjected to qRT-PCR. Each point represents the value from individual sample; horizontal bars show the mean. Results are representative of three independent experiments. Underlying data can be found in S1 Data. BALB/3T3, immortalized BALB/c mice-derived fibroblast; CTMC, connective tissue–type mast cell; IFN, interferon; qRT-PCR, quantitative reverse transcription PCR; WT, wild-type. (TIF) [file pbio.3000530.s001.TIF]

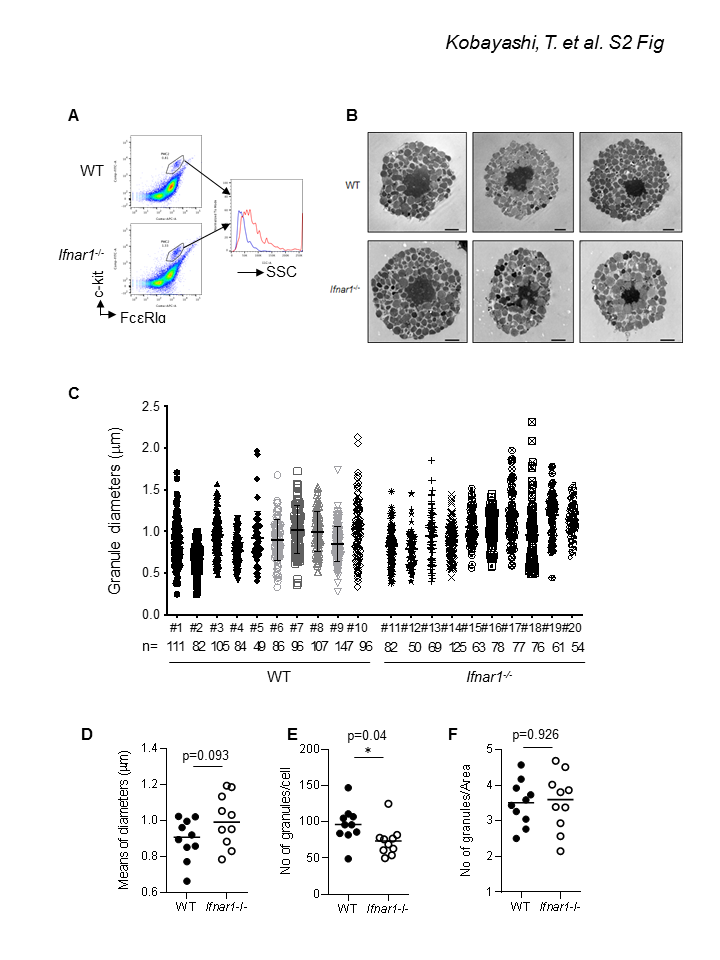

Supplement: S2 Fig — (A) Sorting strategy of peritoneal mast cells from WT or Ifnar1−/− mice. Peritoneal MCs were separated as c-kit+, FcεRIα+ cells on FACSAria II. SSC of the separated mast cells was shown in the histogram. (B) Representative images of WT or Ifnar1−/− peritoneal MCs were obtained by using transmission electron microscopy (TEM). Scale bars, 2 μm. (C) Diameter of the intracellular granules of WT or Ifnar1−/− peritoneal MCs. Sizes of the intracellular granules were determined by measuring the length of the granules on TEM images using ImageJ software. (D-F) Statistical analysis of the character of intracellular granules. (D) Intracellular granule size of WT or Ifnar1−/− peritoneal MCs. Dots indicate individual cells and bars indicate mean values (n = 10, each group). (E) The granule number in a single WT or Ifnar1−/− peritoneal MC. Dots indicate individual cells and bars indicate mean values (n = 10, each group). *P < 0.05. (F) The granule number per the surface area of a single WT or Ifnar1−/− peritoneal MC. Dots indicate individual cells and bars indicate mean values (n = 10, each group). Underlying data from C to F can be found in S1 Data. c-kit, cellular homolog of the transforming gene of a feline retrovirus (v-Kit); FcεRIα, high affinity immunoglobulin E receptor α subunit; MC, mast cell; SSC, side scatter; WT, wild-type. (TIF) [file pbio.3000530.s002.TIF]

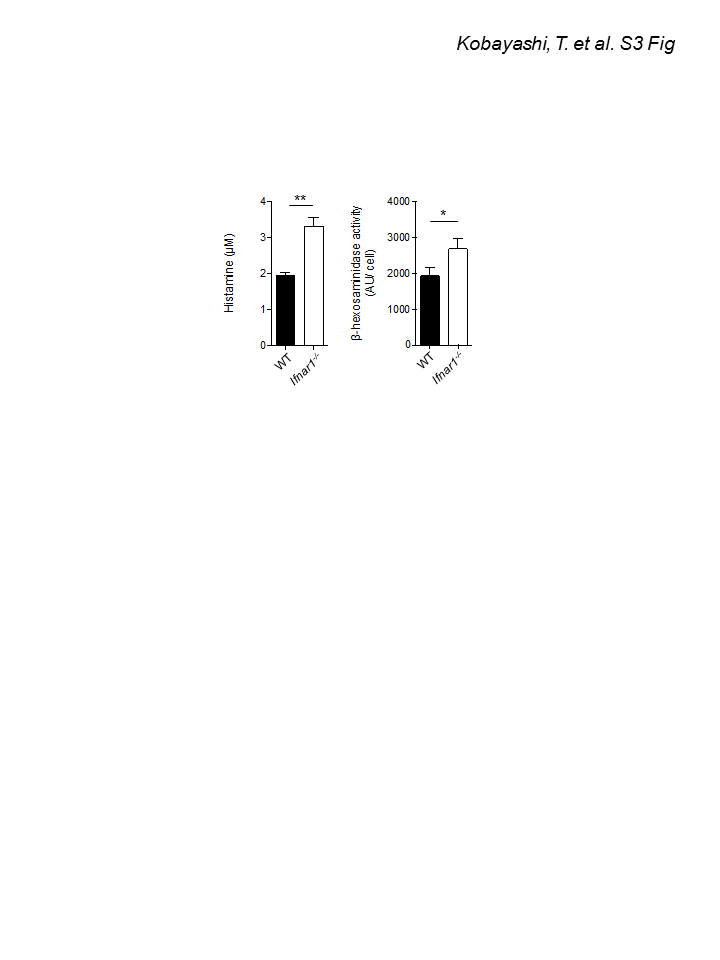

Supplement: S3 Fig — Intracellular histamine level (left) or intracellular β-hexosaminidase activity (right) in WT or Ifnar1−/− mast cells was quantified. **P < 0.01, *P < 0.05, as determined by t test. Results are representative of three independent experiments. Underlying data can be found in S1 Data. CTMC, connective tissue–type mast cell; WT, wild-type. (TIF) [file pbio.3000530.s003.TIF]

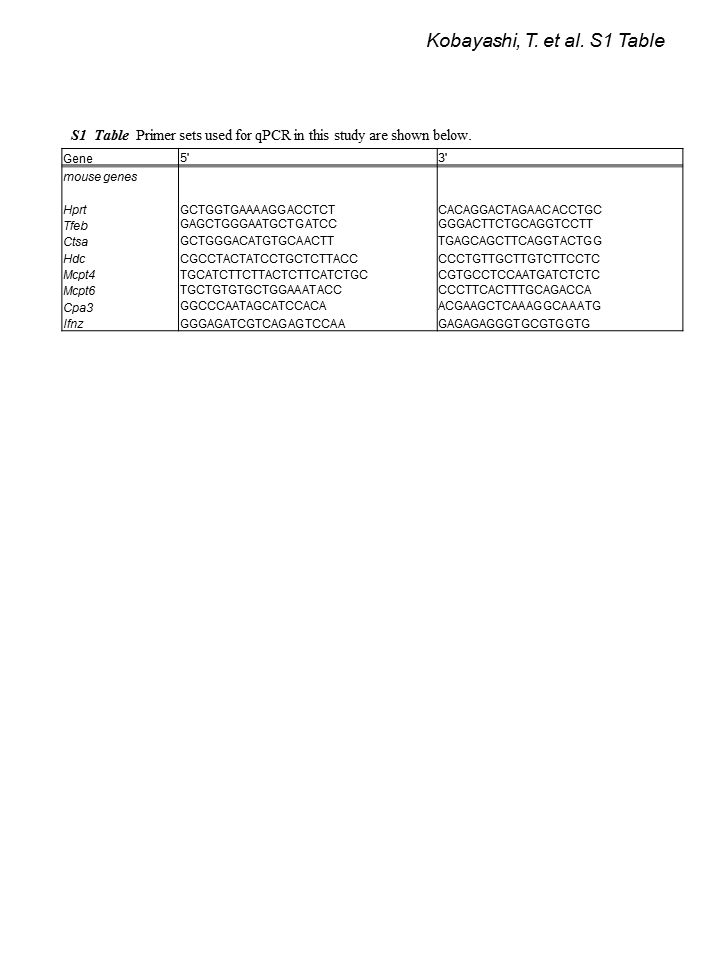

Supplement: S1 Table — qPCR, quantitative PCR. (TIF) [file pbio.3000530.s004.TIF]

Fig 2B

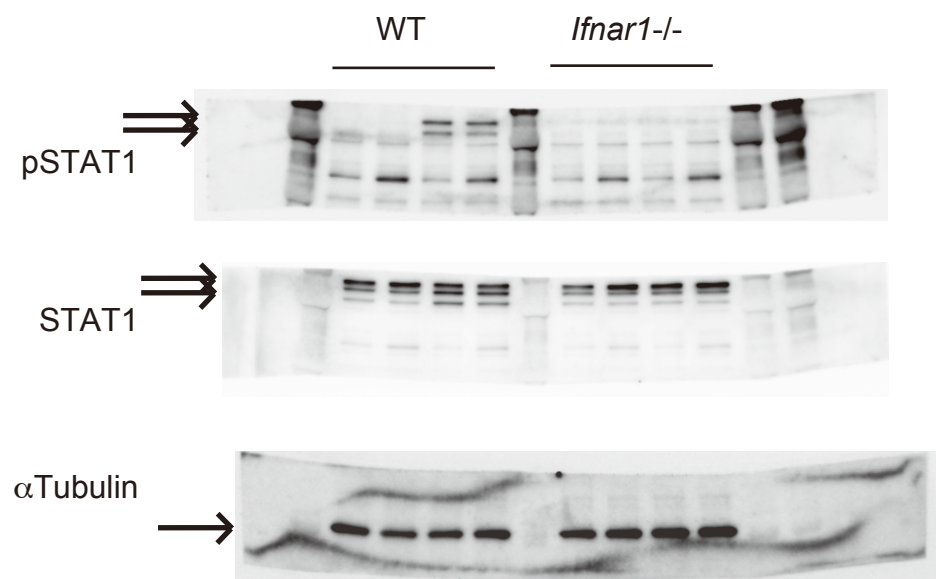

Fig 5A

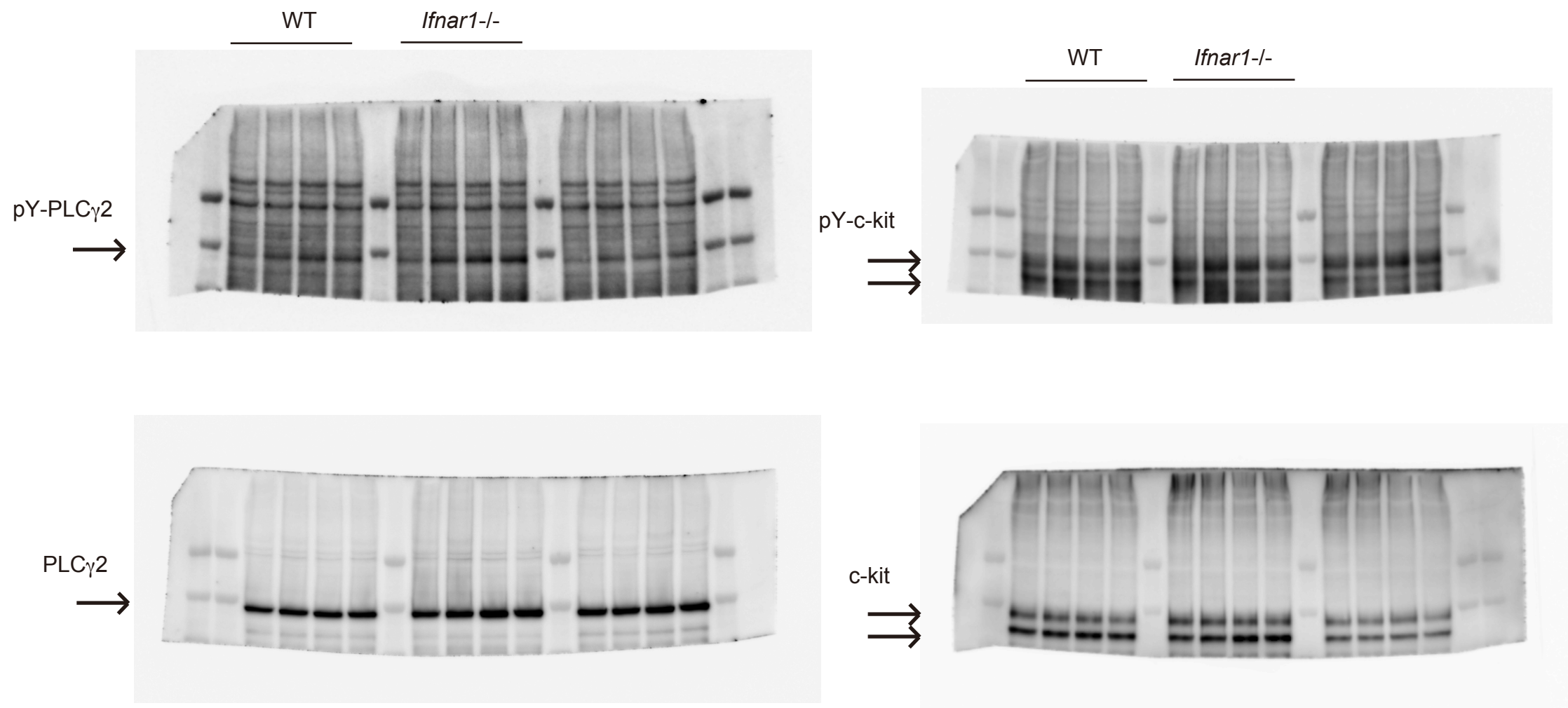

Fig 5A

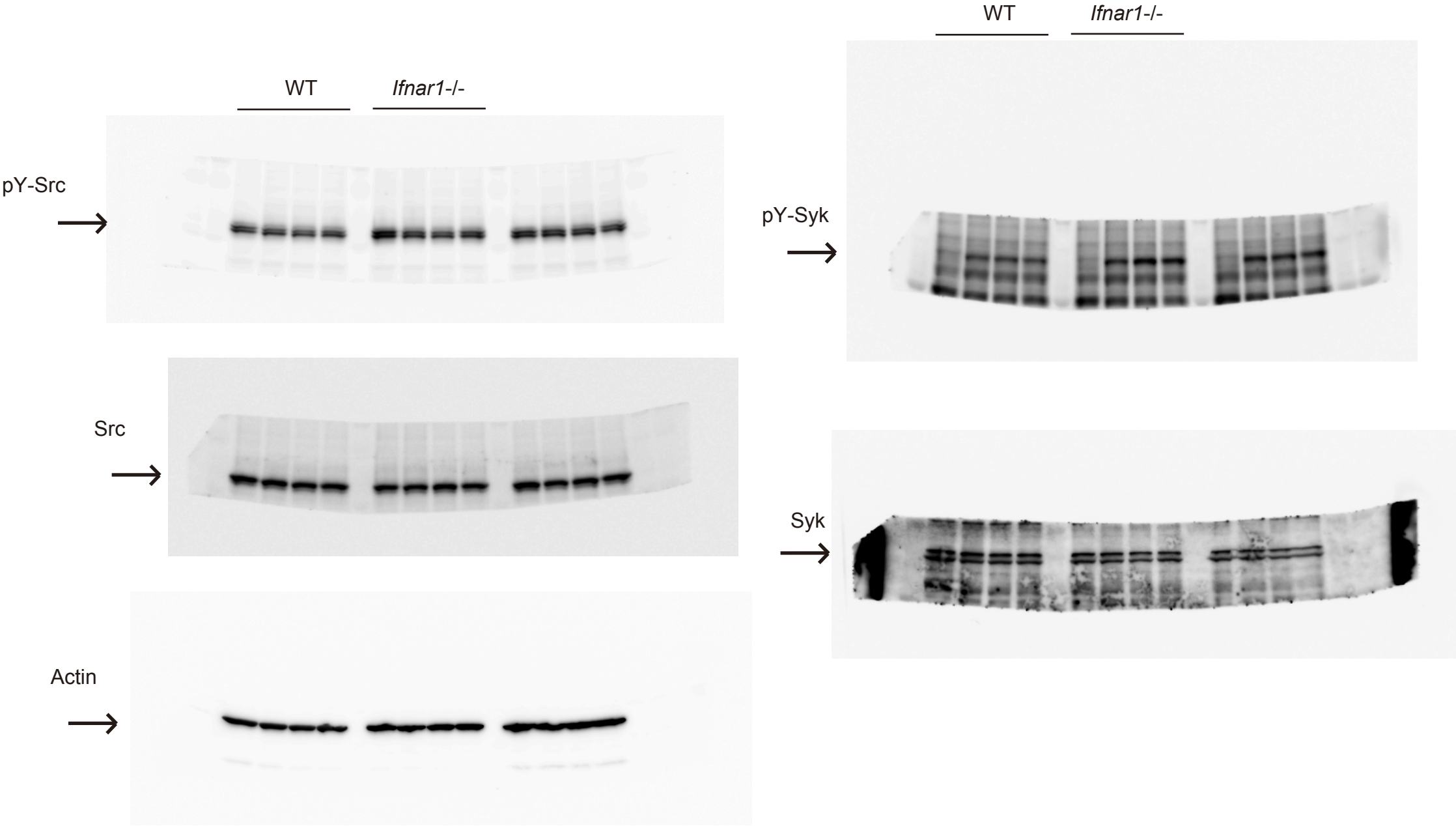

Fig 5B

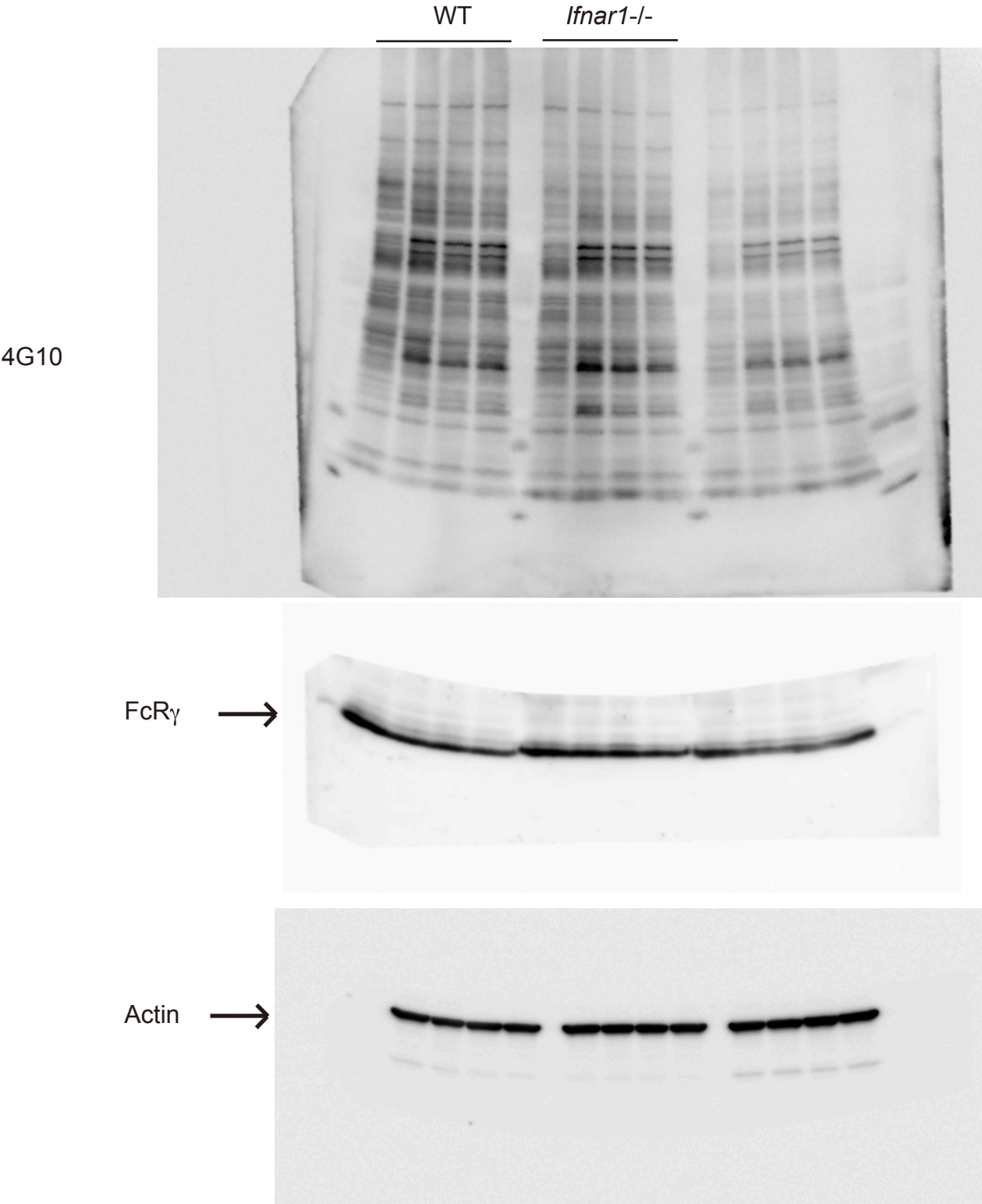

Supplement: S2 Data — (PDF) [file pbio.3000530.s006.pdf]

Fig 7A

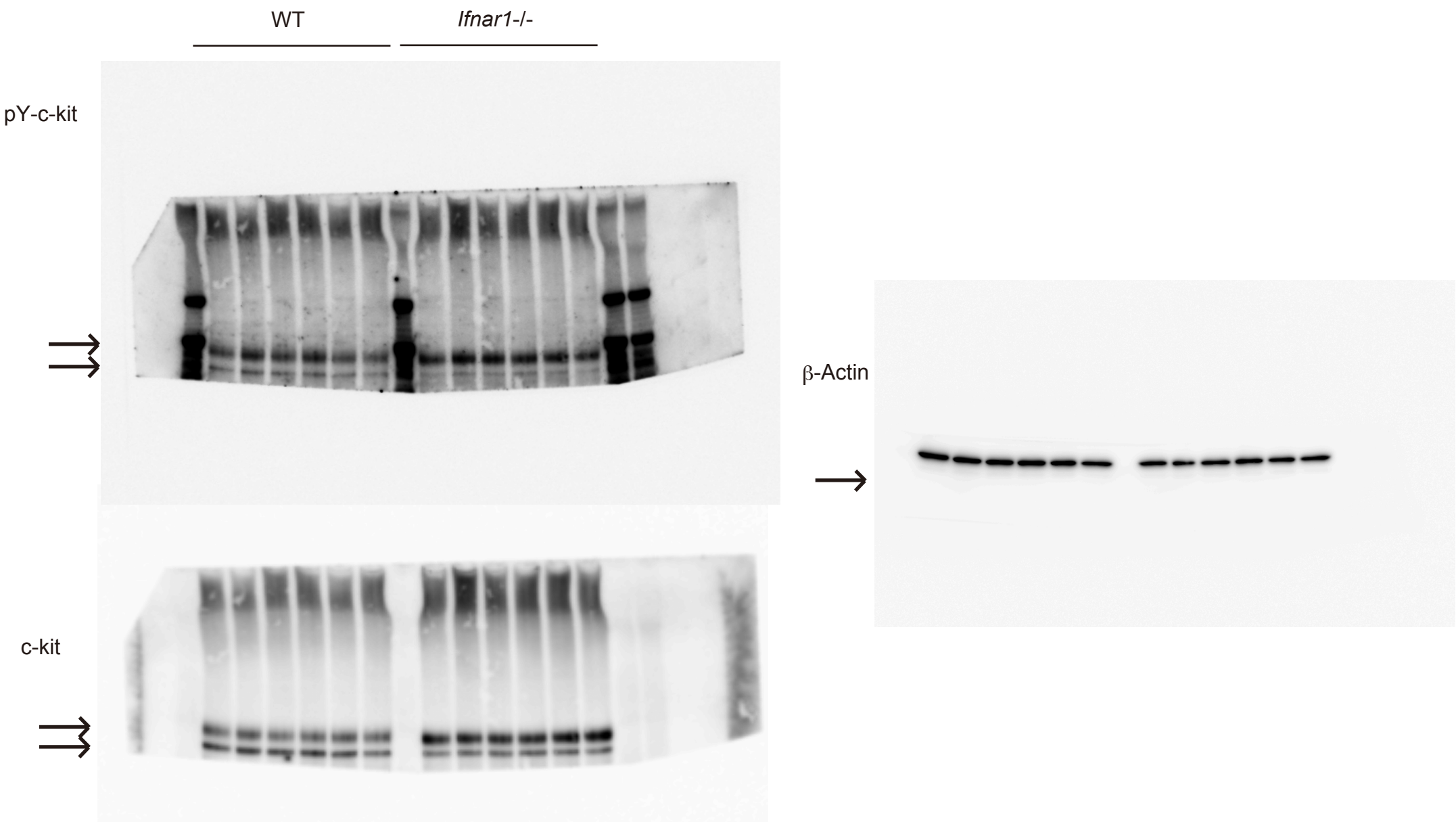

Fig 7A

WT

*lfnar1*<sup>-/-</sup>

pY-  
STAT1

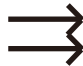

pY-STAT3

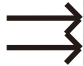

STAT1

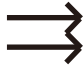

STAT3

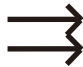

Fig 7A

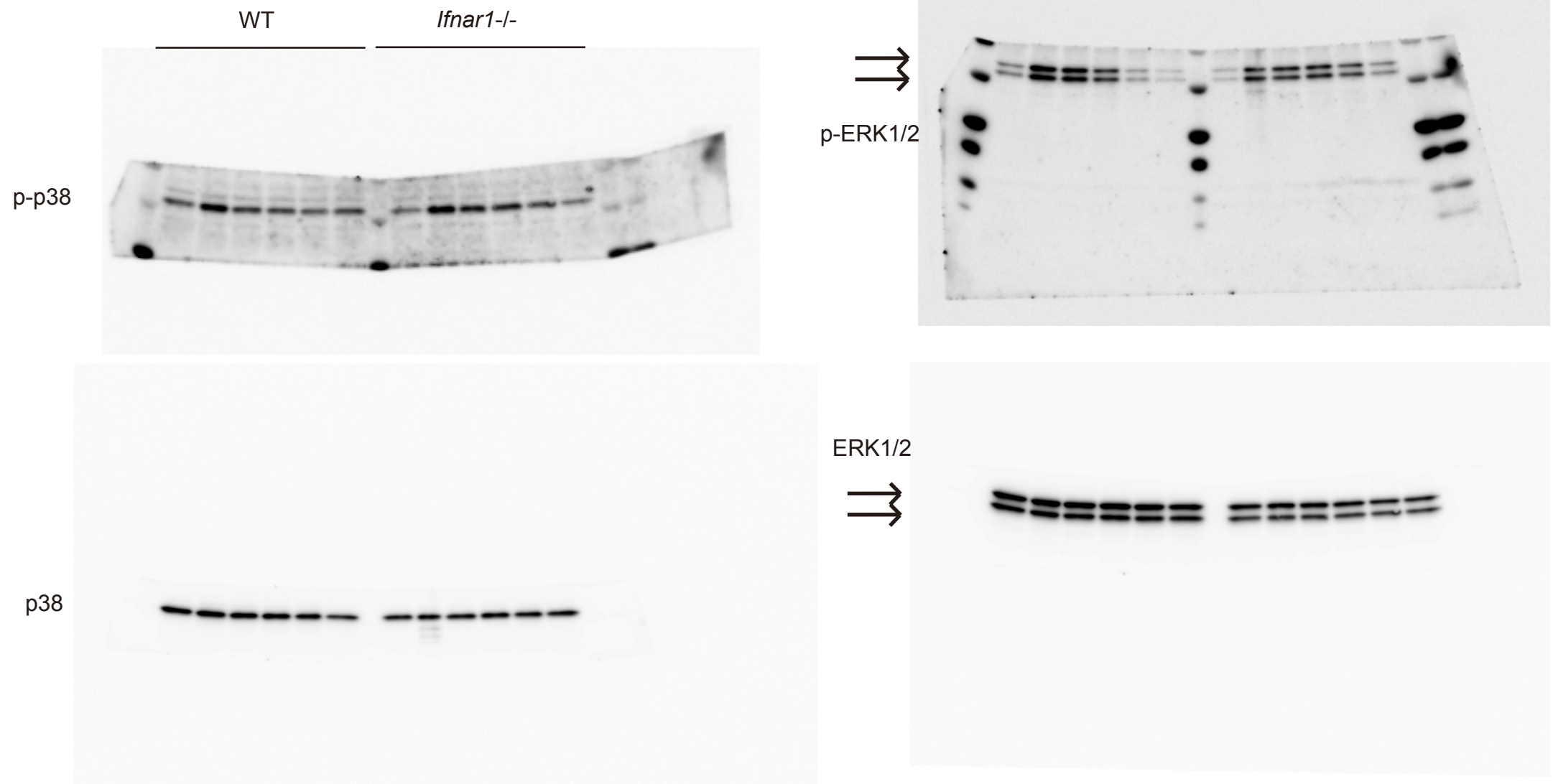

Fig 7B

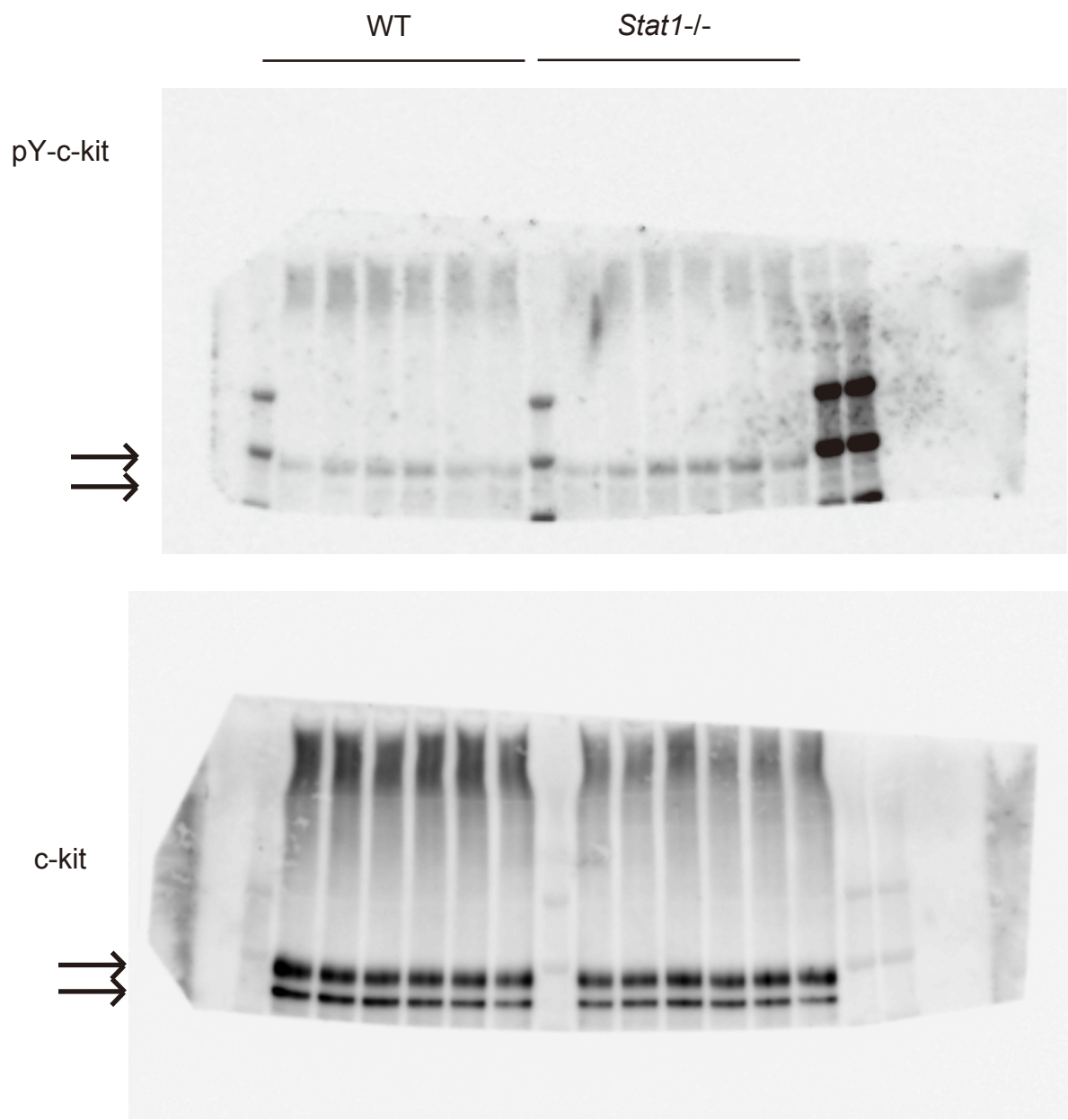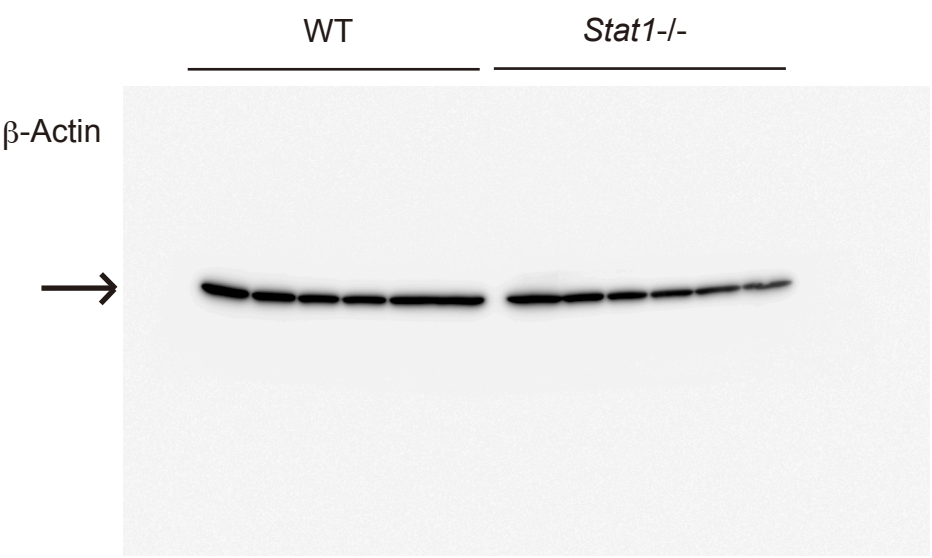

Fig 7B

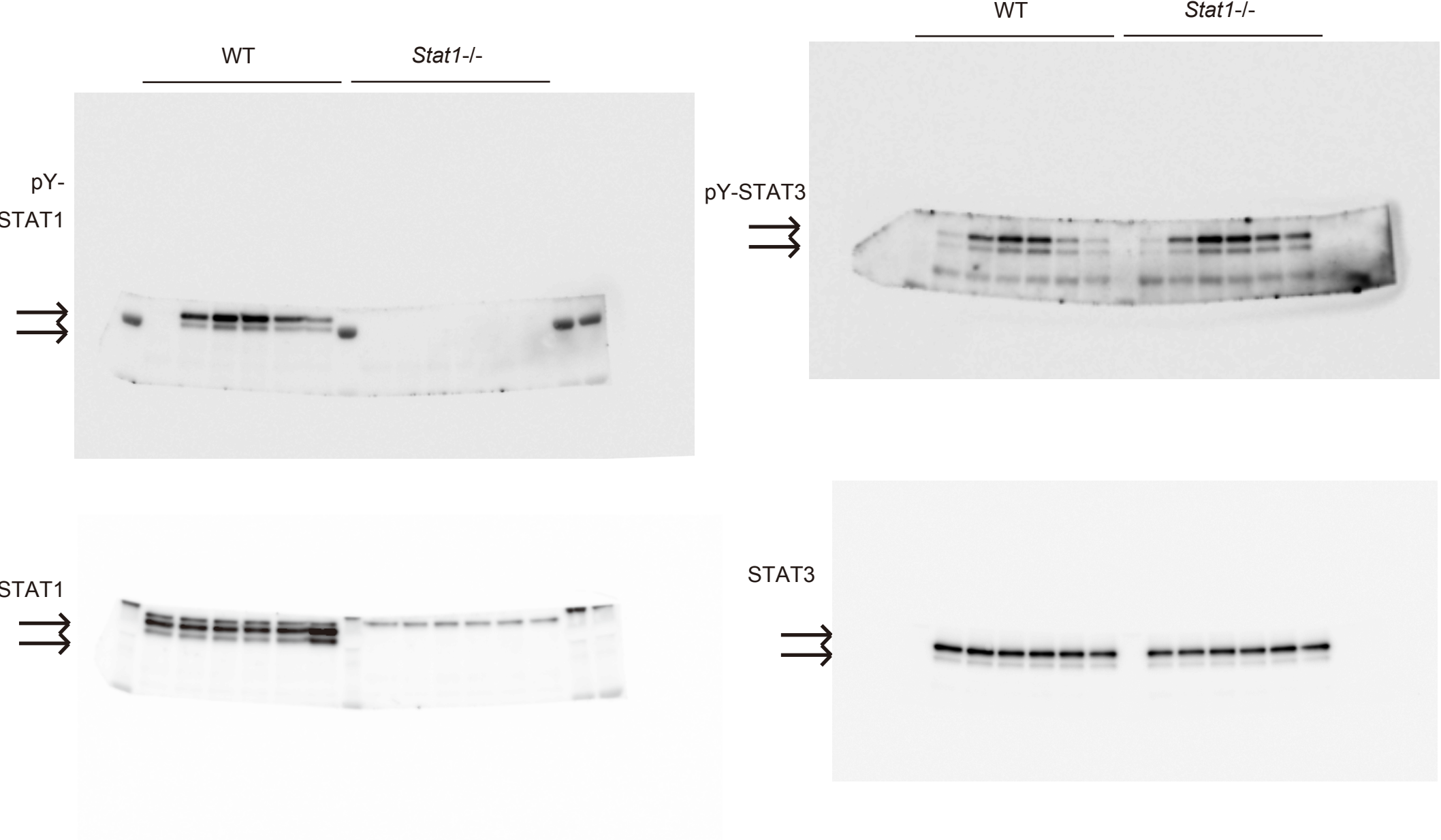

Fig 7B

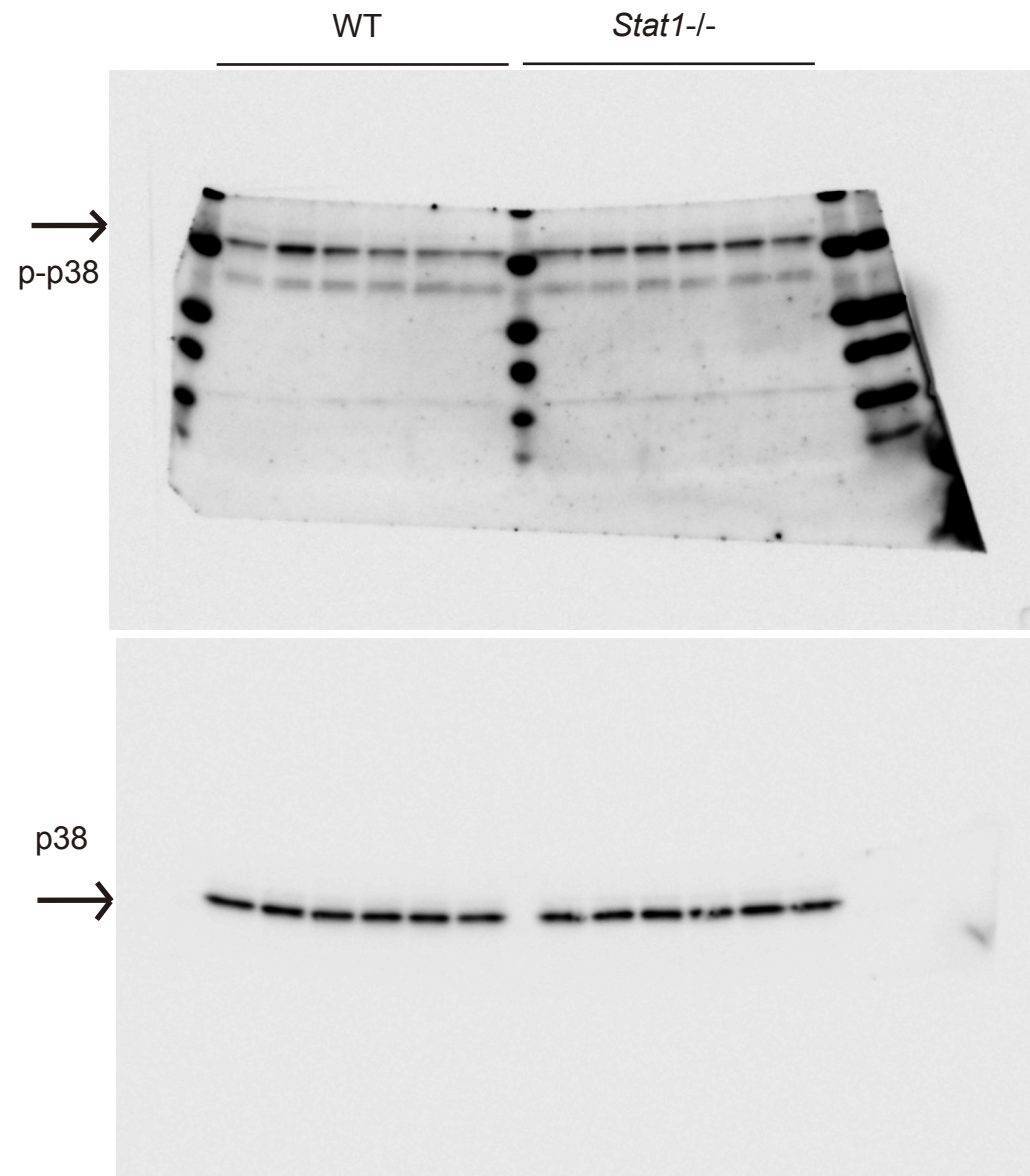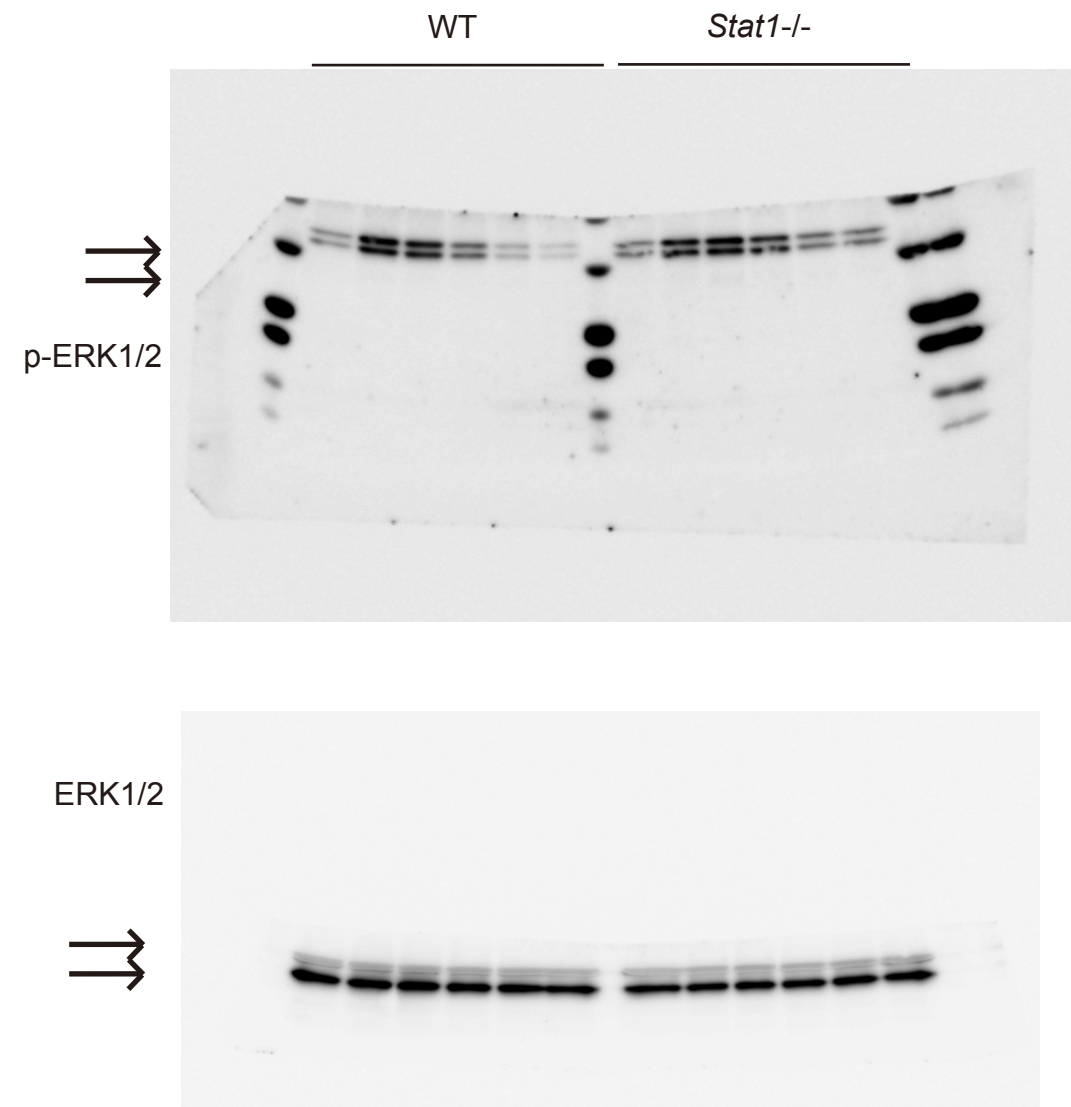

Supplement: S3 Data — (PDF) [file pbio.3000530.s007.pdf]
